# Flubendazole 2.0: Designing a large field trial in dogs for a challenging setting

**DOI:** 10.1371/journal.pntd.0014482

**Published:** 2026-07-02

**Authors:** Amy C. Dupper, Balaji Ramesh, Laura Binkley, Christopher A. Cleveland, Ellen K. Haynes, Guilherme G. Verocai, Meriam N. Saleh, Michael J. Yabsley, Timothy G. Geary, Charles D. Mackenzie, Fernando Torres-Velez, Deborah L. Elder, Wided Najahi-Missaoui, Karmen Unterwegner, Hubert Zirimwabagabo, Métinou Koumétio Sidouin, Philip Tchindebet Oaukou, Bongo Nare Richard Ngandolo, Adam J. Weiss, Rebecca Garabed

**Affiliations:** 1 Division of Epidemiology, College of Public Health, The Ohio State University, Columbus, Ohio, United States of America; 2 Department of Veterinary Preventive Medicine, College of Veterinary Medicine, The Ohio State University, Columbus, Ohio, United States of America; 3 Southeastern Cooperative Wildlife Diseases Study, University of Georgia College of Veterinary Medicine, Athens, Georgia, United States of America; 4 Center for Ecology of Infectious Diseases, University of Georgia, Athens, Georgia, United States of America; 5 Department of Veterinary Pathology, College of Veterinary Medicine and Biomedical Sciences, Texas A&M University, College Station, Texas, United States of America; 6 Warnell School of Forestry and Natural Resources, University of Georgia, Athens, Georgia, United States of America; 7 Institute of Parasitology, McGill University, Ste-Anne-de-Bellevue, Québec, Canada; 8 The Carter Center, Atlanta, Georgia, United States of America; 9 Department of Pharmaceutical and Biomedical Sciences, University of Georgia, Athens, Georgia, United States of America; 10 The Carter Center, N’Djamena, Chad; 11 Programme National d’Eradication du Ver de Guinée (PNEVG), Ministère de la Santé Publique, N’Djamena, Chad; 12 Institut de Recherche en Elevage pour le Développement (IRED), N’Djamena, Chad; University Hospital Bonn: Universitatsklinikum Bonn, GERMANY

## Abstract

Dracunculiasis, or Guinea worm disease (GWD), was targeted for eradication in 1986 after which, the annual incidence decreased by over 99.9%. As human cases of GWD near elimination, dogs have become the primary reservoir for the parasite and remain a challenge for eradication efforts. The high burden of GWD in dogs relative to humans highlights the need for therapeutic interventions in conjunction with behavioral interventions. Laboratory experiments in ferrets demonstrated that flubendazole is partially effective in inhibiting the infectivity of *D. medinensis* larvae. However, the implementation of large clinical field trials in dogs has proven challenging in settings where access to study areas is unreliable throughout the year and attrition is high. Alternative study design approaches are required to address these challenges. This paper outlines challenges encountered during a previous field trial and ways in which these challenges were addressed in the study design and implementation for a second clinical trial of flubendazole in dogs. The results of the second clinical trial are outlined in a separate manuscript.

## Author summary

In 1986, Guinea worm disease (GWD) was formally targeted for eradication, and through behavioral interventions and education programs, the worldwide annual incidence of GWD was decreased from over 3.5 million human cases in 1986 to just 15 cases in 2024. Despite these successes, dogs have emerged as a reservoir for GWD, with Chad experiencing the highest number of dog infections globally in 2023. Large clinical trials in low-resource and remote settings are challenging, and a prior field trial of flubendazole on GWD in dogs did not find a significant effect of the drug, which was attributed to difficulty in finding dogs at follow-up and high attrition. However, the trial was designed to look at individual outcomes in treated dogs within a year after treatment, yet the measurable effect of flubendazole likely occurs at the population level with effects being observed over a longer period of time. Focusing on long-lasting effects of a therapeutic intervention at the population level is somewhat unique in the clinical trial literature and required restructuring the study design including adjustments to sample size, randomization, enrollment, and dose. We outline challenges to this large-scale clinical trial and ways in which we addressed them through study design and study implementation.

## Introduction

*Dracunculus medinensis* is the parasitic nematode that causes Guinea worm disease (GWD) and is transmitted to its definitive host by drinking water containing copepods infected with the third stage larvae (L3) [1–3]. In 1980, eradication of GWD was added as a sub-goal of the United States Centers for Disease Control and Prevention’s (CDC) International Drinking Water Supply and Sanitation Decade initiative due to the understanding that humans were the most common definitive hosts of the parasite, and while animal infections were known to occur, they were rare [4–6]. The Carter Center (TCC) has partnered with ministries of health in endemic countries to lead eradication efforts with support from the World Health Organization (WHO), United Nations Children’s Fund (UNICEF), and other partners since 1986 when there was an estimated 3.5 million human cases in 21 countries [7,8]. The annual incidence of GWD in humans was reduced by over 99.9% through the use of combined targeted prevention measures including increasing access to safe drinking water through the use of bore wells, providing straws with filters to prevent consumption of intermediate hosts, treating bodies of water with an organophosphate compound to control intermediate hosts, community-based surveillance, partnering with local ministries of health, educating communities, incentivizing individuals to report cases of GWD, and containing infected individuals [1,9–11]. In 2024 a total of 15 human cases were reported in two countries: Chad and South Sudan [12].

Despite such success, the complex life cycle of *D. medinensis* adds to the challenge of eradication. First-stage GW larvae (L1) are released into the water where they are ingested by a copepod, the intermediate host [1,13,14]. The larvae undergo two molts within the copepod until it reaches the infective stage (L3) [1], and a mammal must ingest a copepod containing L3s for infection to occur [1,2,14–18]. Digestive juices from the host’s stomach subsequently kill the copepod and release the L3s, after which they migrate to the abdominal cavity, where worms mature into adults and mate within the host [1,14,15]. The male worm dies shortly after mating, while the female worm continues to develop. By month 10 post-infection, the embryos within the female have developed into L1s, and the gravid female worm moves through the host’s subcutis towards the limbs [1, 14, 15]. Immediately before emergence, a painful blister will form at the site as a host response [15,19]. The pain is reportedly alleviated by submerging the affected limb into water, triggering the female worm to release her larvae, where they are then consumed by copepods, thus continuing the life cycle [1,9,13,20,21].

GWD was endemic in Chad until 2000, when the Ministry of Health reported its last human case and transitioned to passive surveillance [21,22]. Human cases re-emerged in Chad in 2010 when GWD was again declared endemic and active surveillance was reinitiated [23]. Following the implementation of the active surveillance program in Chad, *D. medinensis* worms were reported emerging from dogs in 2012 [23]. Since 2012, infections of GWD in dogs have far surpassed human cases and appear to be clustered in villages located along the Chari River [24]. A total of 3,371 dogs were found to be infected, with an average of 1.9 worms per dog between 2015 and 2018 in Chad [25]. An increase in the number of reported GW infections in dogs in Chad was followed by an increase in reported cases in humans, suggesting that dog infections are driving human infections [26]. Additionally, the *D. medinensis* worms that infect humans are genetically indistinguishable from those that infect animals [26,27]. With the highest incidence of GWD occurring in animal hosts, the requirement of eradication of GWD has evolved to now include the interruption of transmission of *D. medinensis* in animals and humans [28].

Dog infections with GW follow a seasonal trend, most often occurring between March and September with dogs having high worm burdens and experiencing recurrent infections [23, 25]. The differing epidemiology of GWD between humans and dogs in Chad points to a novel mode of transmission, with one hypothesis being the consumption of paratenic (amphibians, fish) or transport (fish) hosts [23,25,29]. The seasonality of GWD can be attributed to both the biology of the disease, a 12 month prepatent period, and ecological conditions [15,29]. Peak transmission occurs at the beginning of the wet season when water levels are lowest and fishing activities increase the exposure risk for dogs [29]. Controlling GWD in dog populations is imperative to prevent the parasite from spreading to other populations and to achieve the goal of eradication from human and animal populations. Therefore, beginning in 2014, Chadian villages with a high incidence of GWD were offered a monetary reward to owners to tether their infected dogs to prevent them from contaminating bodies of surface water with GW larvae [7,30]. In March 2020, the Chad National Guinea Worm Eradication Program (identified by its French acronym, PNEVG-T) launched the proactive tethering program (identified by its French acronym, APCC) in villages experiencing endemic GWD transmission [12]. APCC was again expanded in 2020 to include 344 villages [31]. Given the continued infection of dogs, despite the eradication interventions in place, effective therapeutic interventions are needed to ensure there is no continued propagation.

Flubendazole (FLBZ), a benzimidazole anthelmintic drug, was found to impact the embryogenesis and larval development of filarial parasites in humans that cause lymphatic filariasis and onchocerciasis [32,33]. The drug was also shown to be highly effective in treating filarial infections in animals when administered subcutaneously, whereby FLBZ exhibited a slow release over at least three months [34]. Because these filarial nematodes are phylogenetically closely related to *D. medinensis*, as both belong to the suborder Spirurina, efforts were subsequently focused on determining the effectiveness of FLBZ in treating GW infections in dogs. A laboratory trial found that three out of the five ferrets experimentally-infected with *D. medinensis* L3s and then treated with FLBZ (15 mg/kg over three consecutive days) had mature gravid female worms upon necropsy after 10–12 months post-exposure [35]. However, the larvae in the gravid females extracted from treated ferrets exhibited reduced motility in the water, and when exposed to copepods, resulted in < 1% infection rate in copepods compared to a 31% infection rate for larvae recovered from ferrets infected with *D. medinensis* larvae and not treated with FLBZ [35]. Given the encouraging results from the ferret study, a field trial (henceforth “FLBZ 1.0”) of Chadian peri-domestic dogs was carried out in May 2019 until June 2020. Twenty-three villages with a high estimated percent prevalence of GWD in dogs were chosen for inclusion, and half of the dogs in each village were randomized to receive a subcutaneous injection of FLBZ (15 mg/kg for three consecutive days) or placebo every six months [35]. Dogs were followed for one year with the study team examining enrolled dogs for subcutaneous and emerging worms. The overall village level percent prevalence of GW in dogs over the year study period was approximately 6.6%, however, no significant difference in the village-level percent prevalence between the FLBZ-treated and placebo dogs was found [34].

The apparent lack of an effect of FLBZ in domestic dogs in Chad in FLBZ 1.0 was thought to be due to challenges encountered while conducting large-scale studies in resource-limited settings including loss of dogs at follow-up, difficulty accessing portions of the population due to flooding and frequent movement of owners and dogs to inaccessible areas, and high attrition, all resulting in a smaller effective sample size than expected [35]. The authors of FLBZ 1.0 concluded that a larger sample size and a different study design were needed [35].

Another factor was the one-year follow-up time may have been insufficient to capture decreases in emerging *D. medinensis* worms. *D. medinensis* worms are currently only detectable in live mammals when an adult female worm is visually observed or palpated in the subcutaneous tissue or when it emerges from the host 10–12 months post-infection. These worms are only assumed to be *D. medinensis* until they are genetically and morphologically laboratory confirmed. A measurable effect of FLBZ would be observed in individually treated dogs after one year (the first-generation) if the drug impacted the developing *D. medinensis* worm within the host, resulting in fewer emerging *D. medinensis.* However, FLBZ was found to reduce fertility in adult female worms in treated dogs, resulting in fewer viable larvae [32,33,35]. Therefore, the measurable effect of the drug would only be observable in the second-generation, which would require a longer follow-up time.

Based on the results of the FLBZ 1.0 field trial, a second field trial (henceforth “FLBZ 2.0”) was designed to test the effectiveness of FLBZ (a single 100 mg/kg subcutaneous injection vs. three 15 mg/kg subcutaneous injections over three days in FLBZ 1.0) in controlling GWD in peri-domestic dogs in Chad. Careful consideration was given to the development of the study design to address the specific challenges encountered in FLBZ 1.0. Specifically, we chose to look at outcomes at the village level because population-level effects may be more appropriate given that FLBZ was shown to inhibit embryogenesis of L1s, resulting in reduced infectious larvae in local water bodies. Our outcome of interest was the yearly count of emerging *D. medinensis* female worms in subsequent years, which is a more sensitive measure of infectiousness present in a village compared to the number of hosts with emergent *D. medinensis* worms. FLBZ 1.0 experienced significant attrition over the study period, so we have designed the study such that multiple enrollment rounds occured over the study period. This ensured that the proportion of dogs in villages assigned to receive treatment remained high and provided a consistent population-level dose of FLBZ. Lastly, the FLBZ 1.0 trial measured outcomes after 12 months, but FLBZ 2.0 was designed to measure outcomes over 20 months thus allowing us to assess longer-term effects of treatment. Here, we specifically outline 1) the calculation of the required number of treatment and control villages needed to give our study sufficient power to detect a difference in the effect of the intervention at the population level; 2) ensuring equivalence between treatment and control villages by following a restricted randomization in the selection process [36]; 3) ensuring that the location of the selected control and treatment villages followed a spatially random assignment to eliminate spatial autocorrelation or second order effects as well as bias introduced by migration of dogs between villages assigned to different treatment arms; and 4) assuring consistent population-level dose of FLBZ. Finally, we discuss how we addressed implementation and give a high-level overview of attrition and enrollment observed in FLBZ 2.0. A more detailed description of the results from the FLBZ 2.0 trial can be found in a separate manuscript.

## Methodology

### Ethics statement

The FLBZ 2.0 study received approval from the National Bioethics Committee of the Chadian Ministry of Higher Education, Research and Innovation (005/PR/MESRI/SE/DGM/CNBT/SG/2022) as well as the Institutional Animal Care and Use Committee of the University of Georgia (A2019 04 005). Dog owners were informed about study activities and provided verbal and written consent upon enrollment. Assent was confirmed at each follow-up round. In this publication we refer to all distinct inhabited places as “villages” because this is consistent with the terminology used by the Guinea Worm Eradication Program (GWEP) and in prior publications, although many of these places could be called “towns” or “suburbs” to describe their population size and level of development.

### Study area

**Fig 1:**
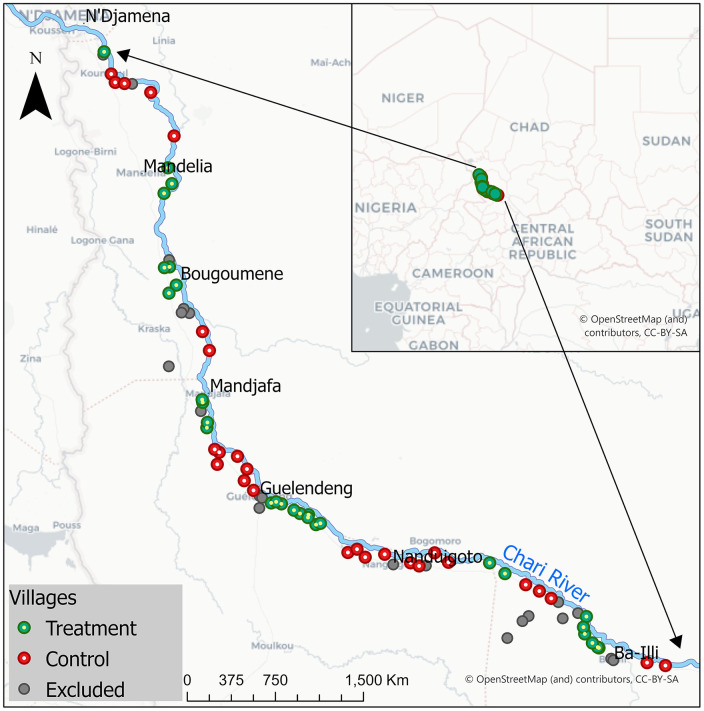
Study area of villages chosen for inclusion into the study and treatment arm assignment. Basemap tiles by CARTO using data from OpenStreetMap contributors (Open Database License, ODbl: https://opendatacommons.org/licenses/odbl). River data from the Humanitarian OpenStreetMap Team (HOT OSM), also available under the ODbl (https://opendatacommons.org/licenses/odbl) [37–39]. CARTO Positron basemap style licensed under CC BY 4.0 (https://creativecommons.org/licenses/by/4.0).

Villages must have had at least one GW infection since 2018, be enrolled in APCC at the time the study began in 2021 and be accessible throughout the year to be eligible for inclusion in the present study. The study area included a total of 75 villages that met the study criteria in N’Djamena, Mayo-Kebbi Est, and Chari-Baguirmi regions located south of N’Djamena, the capital city of Chad, and along the west bank of the Chari River (Fig 1), which also overlapped the study area for FLBZ 1.0 [35]. Because of the differences in exposure to water and surveillance intensity for dogs in APCC versus non-APCC villages, only the 75 villages that had been under active surveillance for dogs since 2018 and were currently enrolled in APCC were eligible for inclusion in the current study. Based on surveillance data provided by TCC and PNEVG-T, a median of 37 (Interquartile Range [IQR] = 56) dogs in the census met our study inclusion criteria. The mean incidence proportion of *D. medinensis* infections in dogs per village within the study area was 11.8% (95% CI: 9.5%, 14.2%), though these dog census estimates included tethered, untethered, and puppies under six months old, not all of which met our study inclusion criteria.

### Exclusion criteria

Villages on the east bank of the Chari River were excluded from the FLBZ 2.0 study because they were difficult to access. In addition, villages that had not had at least one GW infection between 2018–2020 were excluded. Out of the 75 villages eligible for inclusion in the current study, 10 villages with a median of more than 103 dogs (range: 20–476) were excluded given the logistical challenges with surveillance follow-up on such a large number of dogs (Fig 1). Additionally, these 10 villages were centers of the fish trade where residents from surrounding villages would gather on market days to sell fish harvested at different sections of the Chari River and other water bodies [40]. While we were unable to control for the exact route of transmission, excluding these villages reduced the likelihood of including dogs that became infected via consumption of fish acting as a transport or paratenic host originating from a water source outside of their home village. Three villages without any dog counts according to PNEVG-T census estimates were also excluded. Thus, the final number of villages considered for further analysis was 62.

### Proposed statistical analysis

We proposed to measure the impact of treatment with FLBZ in FLBZ 2.0 using a difference-in-differences (DID) study design where our dependent variable was the yearly count of emerging *D. medinensis* female worms per village. The DID analysis specifically measures the average treatment effect on the treated, so the estimate reported from the analysis was the average yearly change in the count of emerging *D. medinensis* female worms in FLBZ-treated villages compared to control villages post-treatment compared to pre-treatment. We compared yearly outcomes at the population level pre-treatment (November 2019 – October 2021) to population level yearly outcomes post-treatment (November 2021 – October 2023).

DID is a quasi-experimental approach that is used to estimate the causal effect of an intervention by comparing the within group variation in the treated group to the within group variation in the control group pre- and post-intervention [41,42]. This method is suited for this study design and study location because there are many variables that cannot fully be controlled for during the study planning and execution, and DID allows us to compare the change in the mean of emerging *D. medinensis* female worms between FLBZ-treated villages and control villages before and after treatment controlling for time-invariant factors or time-varying factors that are group-invariant [41,42]. Studies in similar settings have utilized DID to understand the effect of treatment or policy change on neglected infectious diseases [43,44]. For DID to be a valid method, the assumption of parallel trends, or treated and control groups have a similar trend in the outcome prior to treatment, must be satisfied, which is often difficult to assess [41,42]. We graphed the mean number of *D. medinensis* infections in dogs each year for FLBZ-treated and control villages to visually verify that the parallel trends assumption was satisfied (S1 Fig) [41,42]. The DID method also requires the common shocks assumption be met, which stipulates that any events occurring post-treatment affect treated and control groups equally [45]. While it is difficult to assess whether the common shocks assumption has been violated, villages eligible to participate in this study had similar intervention strategies in place, were all located near the Chari River, and were as similar as possible before randomization to treatment arm.

The change in the mean count of emerging *D. medinensis* female worms was estimated using a Poisson regression model, including a fixed effect for year, which models the trend in the absence of intervention, a fixed effect for treatment, which models differences in the baseline incidence between arms, an interaction between treatment and year, which models the change in the trend for the treated arm due to treatment, and a random effect for village, which models the different baseline incidence for different villages. The DID (interaction term) estimate provides a percentage increase or decrease in the count of emerging *D. medinensis* worms in dogs between treatment and control villages with reference to pre-intervention counts. Using this method, the observational unit was the village, rather than the dog, so the number of villages needed to be selected to have enough power to find a significant effect if one was truly present.

### Village number power analysis

We performed a power analysis using R statistical software (version 4.3.2). First, we fit a mixed-effects Poisson regression model to observed data of the number of emergent *D. medinensis* worms from the eligible villages from 2019 to 2021 with year included as a categorical fixed effect to avoid assuming linear temporal trends and a random intercept for village (using ‘glmer’ function in the *lme4* package [46]). Because post-intervention data (2022) were not available during the study design stage, expected case counts for the post-intervention period were generated based on model-based predictions for 2021, under the assumption that in the absence of intervention, post-intervention outcomes would follow patterns similar to those observed in the most recent pre-intervention year. We then carried out simulations with different numbers of treatment villages (10, 15, 20, 27) randomly selected from the eligible villages with the remaining villages serving as controls. A 50% decrease in emerging *D. medinensis* female worms due to treatment was determined to be clinically significant by the GWEP. Accordingly, for each simulation, a pre-specified treatment effect corresponding to a 50% reduction in cases was imposed in the post-intervention period by reducing the predicted case counts in treated villages by half, while control villages retained their predicted outcomes. The counts in the simulated data were rounded to the nearest integer. For each treatment allocation, we fit our proposed DID regression model to the simulated data 100 times. The number of times the p-value for the interaction coefficient (i.e., the estimated effect of treatment, which was set to be 50%) was less than 0.05 was noted. The simulation using 27 treatment and 27 control villages produced the greatest number of trials (97 trials out of 100) where the interaction coefficient had a p-value less than 0.05. Thus, 27 treatment and control villages would allow us to detect a 50% reduction in emerging *D. medinensis* worms with 5% significance level and 97% power.

### Village selection

Because the treatment and outcome are measured at the village level and the outcome is the count of *D. medinensis* worms emerging from dogs exposed to the water that treated dogs could have contaminated rather than worms emerging from treated dogs, all dogs in treated villages were considered to be treated and all dogs in control villages were considered to be controls. Based on the results of the power analysis, we randomly selected 27 villages within our study area to receive treatment and 27 villages to act as controls, along with another three replacement villages if any of the selected villages were not accessible or refused to participate. We considered two major factors to ensure the correct assignment of villages to study arms: 1) a need to minimize overlap of dogs between arms to prevent dogs from crossing between treatment and control villages and influencing detection of the change in infection counts in either group, and 2) a need to select treatment and control villages with similar environments to ensure comparability considering the study area spans over 232 km encompassing changing environments from the north-west to south-east. Therefore, we sought to ensure that treatment and control villages were randomly distributed while also being far enough apart to minimize the likelihood of dogs crossing between control and treatment villages.

Coordinates of tethered dogs in the study area were not available, therefore, to address non-overlap, we used a buffer around the centroid of each village. Non-tethered dogs within the study area had an overall range of 10.42 km^2^ and a core range of 1.05 km^2^ [47]. Using these estimates, we selected a three-kilometer buffer (28.27 km^2^) around each centroid to determine if villages could be considered non-overlapping. Though dogs eligible for the study were tethered, due to non-compliance and accidental failure of the tethers, dogs were still reported to roam to some extent. Out of the eligible villages, nine villages were found to be non-overlapping, while the remaining villages overlapped with at least one other village. To prevent mega-groupings containing almost all the villages, two “connector” villages were excluded at this step, leaving 60 villages forming a total of 21 groups (Fig 1). All villages within a group were assigned to the same treatment arm (treatment or control) to ensure that there was no overlap between treatment and control villages. There were 92,378 possible combinations of assigning non-overlapping villages and village groups to treatment arms. For each of the above combinations, the number of villages in each arm and number of dogs in each arm were calculated. Among these combinations, 2,902 were identified whereby the number of villages and dogs were nearly identical (within ± 136 dogs, a threshold based on the first quartile of difference in dog counts from all possible combinations) between the two arms.

To account for potential spatial clustering of the treatment arms moving north to south, a join-count test for spatial autocorrelation [48] was conducted to further filter the 2,902 combinations of assignment into treatment/control arms. The join-count test evaluates whether neighboring pairs assigned to the same arm (with neighbors defined using k-nearest neighbors, k = 3, at the village-group level) occur more frequently than expected under spatial randomness. The null hypothesis of the test is that observed spatial arrangement of arms does not differ from what would be expected under random labeling. For each of the 2,902 assignment sets, we computed the corresponding p-value for the join-count test using the *spdep* package in R [49]. Using these generated p-values, 35 assignment sets with p-values for both treatment and control groups greater than 0.9 were filtered out, and one of the remaining assignments was randomly selected as the final treatment and control assignment (Fig 1). The randomly selected assignment included 29 villages that were assigned to the treatment arm and 27 villages that were assigned to the control arm.

## Study implementation

### Exclusion criteria

One village was excluded from the study and replaced by a back-up village that met inclusion criteria due to a report from that village’s leader that they did not have any dogs. Selection criteria for enrollment of dogs within selected villages included dogs in the village that were >1 year old and not currently pregnant. Dogs under one year of age were not included because: 1) given the duration of the *D. medinensis* life cycle, they could not be detected cases or transmit the parasite, 2) APCC does not enroll and tether puppies, and 3) this age group experiences disproportionate mortality. Pregnant bitches were not enrolled because safety of FLBZ has not yet been determined for pregnancy and there is a potential risk to developing fetuses [50]. Pregnant bitches were not enrolled in the control arm for consistency with the treatment arm. While we do not have data on how many pregnant dogs were excluded in each treatment arm, we calculated the mean number of female dogs enrolled in May 2022 per village, which should include those excluded in October 2021, and found no difference between FLBZ and control villages (312 vs. 226 female dogs, p = 0.45).

### Maintaining consistent population-level dose

All non-pregnant, peri-domestic dogs in enrolled villages that were older than one year were eligible for inclusion in the study with the owner’s consent. Research teams worked closely with PNEVG-T field staff called “supervisors” and “technical assistants” in each village to identify eligible dogs and communicate with owners. The supervisors were village residents assigned to monitor dogs in the APCC program and were familiar with the dogs and their owners. Technical Advisors (TA) were assigned to multiple villages and provided support to the supervisors as well as a connection to the broader eradication program. The research teams communicated with the TAs assigned to their villages about the study schedule and what supervisors and owners could expect roughly one month before each field round and then again during the study to provide updates to the schedule and pass requests that owners be present and have their dogs available on the day the team visited the village. The TAs then communicated the information to the supervisors, who communicated the information to dog owners. Authorization to perform research is a structured process involving official approval first from the Ministry of Health and the Ministry of Livestock and Animal Production in N’Djamena followed by engagement at the regional or provincial level, then the peripheral level, and finally the village level. When the teams arrived in each village, they first met the supervisor, with or without the TA (depending on their availability), and the team and supervisor would then meet with the head of the village to receive permission and answer any questions or concerns before proceeding to work on the dogs in each village.

The study began in October 2021 and ended in May 2023, and the seasonal trend of the disease informed the timing of the first FLBZ dose. Dogs were recruited into the study beginning in October 2021 right after peak emergence and when L3s would be present in dogs. Dogs were followed up approximately every three months in February 2022, May 2022, September 2022, November 2022, and May 2023 with additional recruitments occurring in May 2022 and November 2022 in order to maintain the population dose of FLBZ. Each recruitment round lasted approximately four weeks, and study teams spent approximately one day in each enrolled village recruiting dogs that were identified and tethered through APCC. Enrolled dogs were administered a subcutaneous microchip at the time of enrollment to allow for consistent identification at each follow-up. At each enrollment round, dogs located in villages assigned to treatment were given a single subcutaneous injection of FLBZ (100 mg/kg) specifically formulated to form a depot that was expected to slowly release FLBZ over an extended period of time (up to 6 months). All dogs enrolled in the study in both treatment and control arms were administered a rabies vaccine at the time of enrollment into the study.

Study teams followed up on each of the enrolled dogs approximately every three months beginning in October 2021 and ending in May 2023. At each follow-up, consent was confirmed, dogs were given a physical examination whereby any subcutaneous or emerging worms were documented and reported to the supervisor, the GPS coordinates of the dogs’ locations at the time of data collection were recorded, and additional samples for associated projects were collected [51,52]. Prior to each follow-up, a day-long training session was held in N’Djamena led by trained veterinarians at which time team members were trained on dog handling, methods to detect a subcutaneous or emerging GW, how to consistently document data, and any specific procedures for associated projects that were being done during that round. Outcomes were collected independently by PNEVG-T as part of their regular active surveillance program. PNEVG-T only recorded *D. medinensis* infections if the worm was actively emerging from the host; blisters and lesions were not recorded as infections. Upon emergence, the worm was visually identified and extracted by PNEVG-T staff, and a piece was sent to CDC and Vassar College where microscopic identification and sequencing were, respectively, performed to verify the worm was *D. medinensis*. Regular visits by the study team were designed to monitor the population-level “dose” of FLBZ to make sure that treated and control dogs were present and to track them if they moved. Re-enrollments and treatments were designed to enroll and treat new dogs (or newly eligible dogs) to maintain this consistent population-level dose in a setting known to have a high attrition rate [35], so that there would not be fluctuations in treatment effects.

### Analysis of population-level dose

Analysis of outcome and exposure data was not planned at the individual level, so individual-level attrition was not as crucial to this analysis as to the FLBZ 1.0 study. However, the DID analysis assumes consistent population-level “dose” of FLBZ to treatment arm villages and common overall population trends related to GW between arms (the common-shocks assumption) [53]. To measure the population dose, we used the proportion of dogs enrolled in the study compared with the observed number of dogs in each village, and to measure common shocks, we compared overall attrition between arms.

To assess the proportion of dogs present in each village that were enrolled in the study, we compared the numbers of dogs that our study teams enrolled to the official dog census numbers available through a data request for PNEVG-T’s data. Dog census numbers are updated at variable frequencies across villages, so in cases where multiple numbers were available per year, we took the median of the available estimates. The method used in creating the dog census was inconsistent during the time periods we requested, so some estimates may have included puppies and others may not. None of the census methods used a formal sight-re-sight or other protocol to estimate numbers of unowned dogs.

To compare attrition between the two arms of the study, we included attrition data on each follow-up round of the study. If a dog was not present, the team asked the owner and supervisor what happened to the dog and obtained as many details as possible to assess whether the owner and dog moved away to a village not in the study, the dog ran away or otherwise disappeared, the dog died due to illness, the dog died due to accident, or the dog was killed. Dogs and owners who moved to villages in the study were added to that village’s enrollment and followed there instead of being included in the attrition. If a dog was reported in the attrition on one round of follow-up, the dog’s loss and reason for the loss were confirmed at the next two follow-up visits. Another challenge encountered in the field was the identification of live dogs present when they previously had been reported as lost or dead. Any dogs reported as lost or dead but who were later found alive were not included in the attrition counts. The attrition was described in a series of line graphs and the comparison of survival rate between arms was made using a log-rank test.

## Results

### Population-level dose

The proportion of dogs enrolled and alive per village compared to the official census is reported in Table 1. The proportion varied from 4.2% to 4000% with a median of 50.0%, 53.3%, 49.4% for October 2021, May 2022, and November 2022 enrollments, respectively.

**Table 1:** Census population estimates, number of dogs alive and enrolled, and the proportion of dogs alive and enrolled in October 2021, May 2022, and November 2022.

	October 2021			May 2022			November 2022		
Village	Census pop	# of dogs	% of dogs	Census pop	# of dogs	% of dogs	Census pop	# of dogs	% of dogs
Aligara Sara	15	8	53.3	9	11	122.2	9	18	200.0
Bandama	22	17	77.3	23	18	78.3	23	20	87.0
Bere Ville	130	24	18.5	86	45	52.3	86	42	48.8
Boctole Foulbe Ville	19	9	47.4	17	14	82.4	17	14	82.4
Boudanassa Bao	27	18	66.7	38	20	52.6	38	18	47.4
Boudanassa Lambe	59	20	33.9	68	33	48.5	68	39	57.4
Boudanassa Massa	16	9	56.3	32	11	34.4	32	10	31.3
Boudanassa Sara	40	20	50.0	51	23	45.1	51	24	47.1
Boudouloum	32	30	93.8	97	36	37.1	97	35	36.1
Bougoumene	96	28	29.2	56	36	64.3	56	32	57.1
Boye	48	24	50.0	97	41	42.3	97	38	39.2
Daby	2	8	400.0	1	11	1100.0	1	10	1000.0
Darda	25	21	84.0	26	22	84.6	26	19	73.1
Diamra	592	25	4.2	661	67	10.1	661	77	11.6
Diganali^*^	74	22	29.7	75	40	53.3	75	29	53.3
Djanta Marba	89	32	36.0	158	79	50.0	158	98	62.0
Djanta Sara	82	28	34.1	163	47	28.8	163	54	33.1
Dogoré	87	62	71.3	115	78	67.8	115	86	74.8
Gadaban	18	8	44.4	17	13	76.5	17	10	58.8
Garwaye	220	44	20.0	117	77	65.8	117	53	45.3
Godogo	18	15	83.3	15	13	86.7	15	10	66.7
Gole Nanguigoto	62	34	54.8	63	41	65.1	63	34	54.0
Gomba	23	10	43.5	50	29	58.0	50	12	24.0
Goudoum Goudoum Massa	8	9	112.5	21	14	66.7	21	15	71.4
Goum	12	5	41.7	21	11	52.4	21	9	42.9
Goussou	13	14	107.7	46	15	32.6	46	11	23.9
Kakale Massa	19	14	73.7	25	17	68.0	25	16	64.0
Kakale Mberi	39	38	97.4	127	43	33.9	127	44	34.6
Kelengue^**^	43	46	107.0	132	59	44.7	132	59	44.7
Kolemara Sara	23	11	47.8	38	20	52.6	38	22	57.9
Koundoul 2	58	19	32.8	96	39	40.6	96	37	38.5
Largana	53	37	69.8	110	48	43.6	110	40	36.4
Lobobo	9	6	66.7	13	9	69.2	13	6	46.2
Loumia Centre	516	85	16.5	425	233	54.8	425	231	54.4
Mabaye	10	5	50.0	14	10	71.4	14	9	64.3
Madjiri	3	1	33.3	1	1	100.0	1	1	100.0
Magara 1	15	8	53.3	15	10	66.7	15	10	66.7
Magara 2	6	1	16.7	9	7	77.8	9	8	88.9
Magrao	132	17	12.9	102	23	22.5	102	26	25.5
Mainde	13	13	100.0	26	16	61.5	26	11	42.3
Mandjala	42	22	52.4	64	36	56.3	64	36	56.3
Mede 3 Sara	7	6	85.7	3	6	200.0	3	5	166.7
Medegue	208	27	13.0	108	40	37.0	108	46	42.6
Midjoue	16	13	81.3	24	16	66.7	24	23	95.8
Mogrom Biao	118	30	25.4	56	27	48.2	56	21	37.5
Mogrom Centre	60	8	13.3	39	20	51.3	39	18	46.2
Morgague	152	40	26.3	75	40	53.3	75	36	48.0
Mourai Houssa	1	40	4000.0	128	50	39.1	128	34	26.6
Moursal	61	29	47.5	92	50	54.3	92	46	50.0
Mouskougou 1	27	16	59.3	62	22	35.5	62	13	21.0
Ngara	19	23	121.1	142	48	33.8	142	46	32.4
Ngargue	158	42	26.6	263	99	37.6	263	84	31.9
Raf Centre	66	28	42.4	54	35	64.8	54	31	57.4
Roltang	22	8	36.4	36	19	52.8	36	18	50.0
Sawata	102	19	18.6	55	37	67.3	55	29	52.7
Soukoua	9	8	88.9	19	10	52.6	19	7	36.8

* This includes dog census counts from Mayo-Kebbi Est, Guelendeng, Largana, Diganali and Mayo-Kebbi Est, Guelendeng, Nanguigoto, Digangali

** There are several villages with this same name. These census counts come from Mayo-Kebbi Est, Guelendeng, Largana, Kelengue.

### Attrition

**Fig 2:**
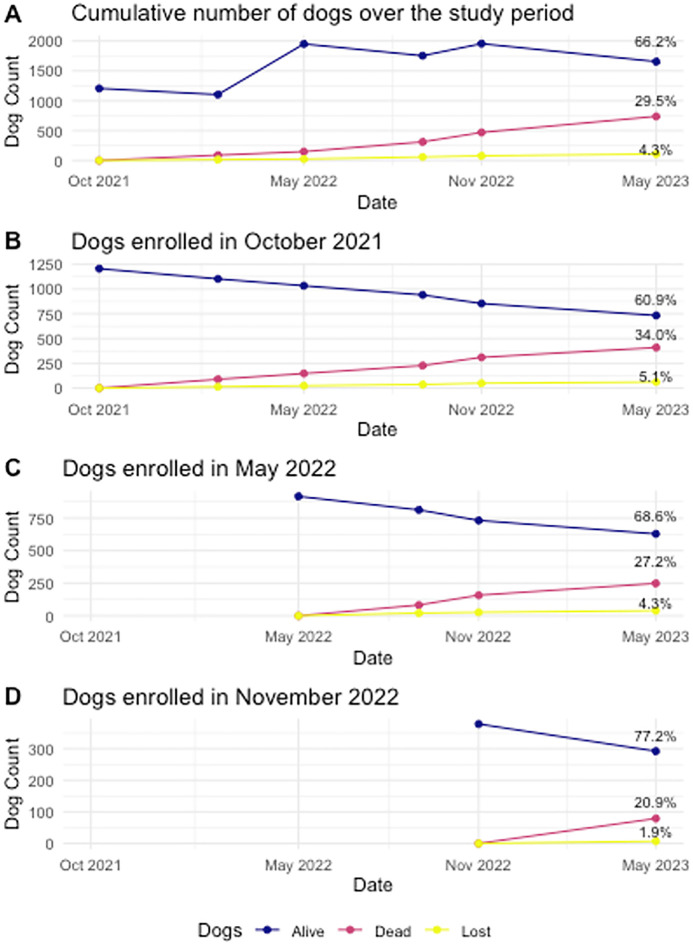
Attrition of enrolled dogs over the study period and by enrollment round.

The FLBZ 2.0 study enrolled a total of 2,501 dogs in the study throughout the study period, October 2021 to May 2023 (Fig 2). Overall, 108 (4.3%) of dogs were lost to follow-up and 738 (29.5%) died during the study period. When looking at each enrollment group individually, there were a total of 1,206 dogs enrolled in October 2021, of which 62 (5.1%) were lost to follow-up and 410 (34.0%) died over the study period. In May 2022, 917 dogs were enrolled, of which 39 (4.3%) were lost to follow-up and 249 (27.2%) died. Finally, 378 dogs were enrolled in November 2022, and 7 (1.9%) were lost to follow-up and 79 (20.9%) died. Additionally, no difference in survival by treatment arm was found overall (Hazard Ratio (HR) = 0.9, 95% CI = 0.8-1.0, *P* = 0.16) or when looking at the enrollment rounds individually in October 2021 (HR = 0.8, 95% CI = 0.7-1.0, *P* = 0.07) or in May 2022 (HR = 0.8, 95% CI = 0.6-1.1, *P* = 0.12). However, dogs enrolled in November 2022 that were randomized to the treatment arm had a lower rate of survival compared to dogs randomized to the control arm (HR = 1.7, 95% CI = 1.0-2.9, *P* = 0.04).

In 29 instances (29 different dogs), the microchip that was subcutaneously implanted in each dog at enrollment was not picked up by the scanner at follow-up. In this case, the dog’s identification was verified by vaccination card or visual confirmation by one of the team members or village supervisors and a new microchip was inserted. We identified 89 dogs who were marked as lost or dead at one follow-up but were later found alive and present. We have included detailed results from the DID analysis in a separate manuscript.

## Discussion

In this paper, we described our novel study design for evaluating the impact of treatment with FLBZ on the average number of emerging *D. medinensis* gravid female worms among per-domestic Chadian dogs per village. This was based on the assumption that FLBZ would act on *D. medinensis* fecundity and impact the second-generation of emerging worms in dogs exposed to larvae from treated dogs. Our study design attempted to address challenges encountered in the FLBZ 1.0 field trial. FLBZ 1.0 enrolled 23 villages and treated half of dogs in each village with FLBZ (N = 222) or a placebo (N = 213), and they measured the prevalence of *D. medinensis* infection in FLBZ-treated and placebo dogs. The current study enrolled a total of 2,501 dogs over three enrollment rounds and measured outcomes at the village level instead of at the individual dog level. The challenges informed by FLBZ 1.0 were based on measuring the outcome, study design (particularly sample size and assignment to treatment arms), and implementation to maintain consistent dose and common shocks across treatment arms.

The primary challenge relating to the outcome was the possibility that FLBZ does not impact the molting of larvae (L3- adult) in treated dogs but rather causes female worms in treated dogs to have fewer viable (L1) larvae [35]. That is female *D. medinensis* worms in dogs treated with FLBZ would still emerge and L1s would be released into the water, but these L1s would exhibit reduced ability to infect copepods. Therefore, mammalian hosts drinking from bodies of water would have a lower probability of consuming copepods with infectious L3s. Instead of observing a reduced incidence in FLBZ-treated dogs after 12 months compared to control dogs, we would expect to see a reduced incidence in all dogs regardless of treatment receipt in a FLBZ-treated village beginning 24 months after treatment compared to dogs in control villages due to a reduction in transmission potential. This was our rationale for looking at the population level outcome of emerging female worms per village as reported in the PNEVG-T active surveillance program and assigning treatment or control to the whole village. Additionally, we followed the dogs for two years instead of one year to better account for the theorized second-generation effect.

In looking at this village-level outcome of treatment on the entire village, we were able to maintain a sufficiently powered sample size of 27 + villages per study arm to detect a 50% or greater difference in number of emerging worms due to treatment in the context of other control programs. However, when using this outcome treatment assignment, we had to consider that adjacent villages were assigned to the same study arm to prevent spillover of the treatment effects to adjacent control villages and vice versa. Our process of using spatial buffers based on untethered dog home ranges and clustering of villages to assign treatment attempted to mitigate this. A future geospatial analysis of GPS points of individual dogs during follow-up could be used to assess the success of the process in mitigating spillover of treatment effects.

In addition to concern for spillover of treatment effects to adjacent villages, we were also concerned with assigning village clusters to study arms without creating spatial autocorrelation in assignment (i.e., all treatment villages clustered in the north and all control villages clustered in the south, for example) and with other assignments that would decrease comparability of the villages in the two treatment arms. To mitigate this concern, we removed arm assignments that had disparate total numbers of dogs or linear clustering of arms from consideration. To assess spatial clustering, we were able to use a runs test due to the linear nature of our study villages along the west bank of the Chari River. In a less linear population, a different spatial test would have been required. Our process of thinning non-comparable arm assignments was a somewhat unique way to maintain random assignment without naively using a biased assignment.

In the implementation of the study, we attempted to mitigate effects of individual dog attrition on the number of FLBZ treatments received by a village by having two additional enrollment rounds throughout the study period as well as regular follow-up of dogs. The top line in Fig 2A indicates that we were largely successful in maintaining a constant number of dogs enrolled in the study, even with a 33.8% attrition rate over the 2-year study. The data in Table 1 suggest that our population dose of FLBZ may have varied widely across villages, which would be concerning. However, there are several possible explanations for the variation. The dog census did not follow a consistent method during this period, so it may be that the census numbers were simply inaccurate. Additionally, names of inhabited places in this area were not as specific as they would appear to be, so names used by the PNEVG-T may have varied within the program over time and the names we matched to the names of our study villages may not have aligned perfectly with the physical location boundaries used in the field. So, though the proportion of dogs enrolled in the study does indicate that the dose of FLBZ administered to villages may have varied over time and village, the proportion of dogs enrolled was not associated with study arm or study team.

Regarding common shocks across study arms, only those dogs newly enrolled in November 2022 had significantly different survival between treatment arms. This could be attributed to an outbreak of canine distemper virus that occurred and was still producing a few cases during the final follow-up period in May 2023. We counted deaths and found the number of deaths suspected to be from canine distemper virus occurred primarily in FLBZ-treated villages. However, we did not track the outbreak, nor did we perform diagnostics on all dogs displaying symptoms of canine distemper virus. Related to this, the dogs lost to follow-up from the November 2022 enrollment did not have their loss confirmed twice given that they were only visited again in May 2023. If the canine distemper outbreak caused differential mortality, we were not able to confirm the death at subsequent follow-ups. Therefore, some of the dogs assumed to be lost or dead may have returned if they had been visited again. The overall attrition in FLBZ 2.0 was slightly improved compared to the 42% over 12 months observed in FLBZ 1.0 [35]. Similar to FLBZ 1.0, we relied heavily on the knowledge of village supervisors and used microchips to more accurately identify individual dogs on follow-up and track attrition.

One limitation of our study design was the number of factors that we could consider in the process of village and group selection. For example, it may have been beneficial to consider the distance of each village to the Chari River, or other large bodies of water, considering the critical role that the aquatic environment plays in *D. medinensis* transmission. However, establishing equivalency with respect to factors other than treatment and control is challenging and would limit the amount of randomization possible. Another limitation was the lack of distinct boundaries for each village, which are typically formed by community grouping and can change dynamically. Land use cover maps generated using high resolution satellite imagery and providing ranges for bodies of water, settlements, and GPS coordinates of dogs may be a helpful next step in the creation of boundaries for villages and to quantify bodies of water within and around these communities. Such quantified metrics would enable future analyses to account for these factors analytically in the models looking at dogs potentially exposed to larvae birthed from treated and control dogs. Our outcome measured emerging worms, but not all infected dogs have an emerging worm, so our estimates most likely underestimate the true incidence of GW infections in dogs. Lastly, the FLBZ 2.0 study design does not account for dogs in villages adjacent to the study villages, which are currently not under surveillance, or free-roaming dogs that visit study villages. The influence of dog movement from unobserved villages may bias the expected effect of treatment in villages enrolled in the study. Our process of assuring that treatment and control arms were not spatially correlated helps to support the assumption that the influence of unenrolled villages would be independent of assignment to treatment or control.

### Future recommendations

Guinea worm eradication presents interesting challenges in the use of therapeutic or preventive (chemoprophylaxis) medications in a true One Health context whereby improved health outcomes relate not only to the treated individuals, but to the surrounding environment that human and animal populations are exposed to. We presented novel methodology and findings that may be useful for similar field trials for other One Health interventions. The FLBZ 1.0 and 2.0 studies highlight the challenges faced when implementing a large-scale randomized clinical trial in an under-resourced setting and an iterative approach to assessing effectiveness. Though challenges still exist in FLBZ 2.0, the study design is consistent with practical large-scale field implementation and should give a more realistic measure of what would be observed if FLBZ were administered to dogs in the APCC program.

## Supporting information

S1 Fig Plot of the mean count of emerging *D. medinensis* worms per year for treatment and control villages between 2019 and 2023. (DOCX)
