# Supplementary material for: Flubendazole 2.0: Designing a large field trial in dogs for a challenging setting
Source: PLoS Negl Trop Dis. 2026 Jul 2;20(7):e0014482. doi: 10.1371/journal.pntd.0014482 (PMC13345460; doi:10.1371/journal.pntd.0014482)

Supplemental Information

S1 Fig. Plot of the mean count of emerging *D. medinensis* worms per year for treatment and control villages between 2019 and 2023.


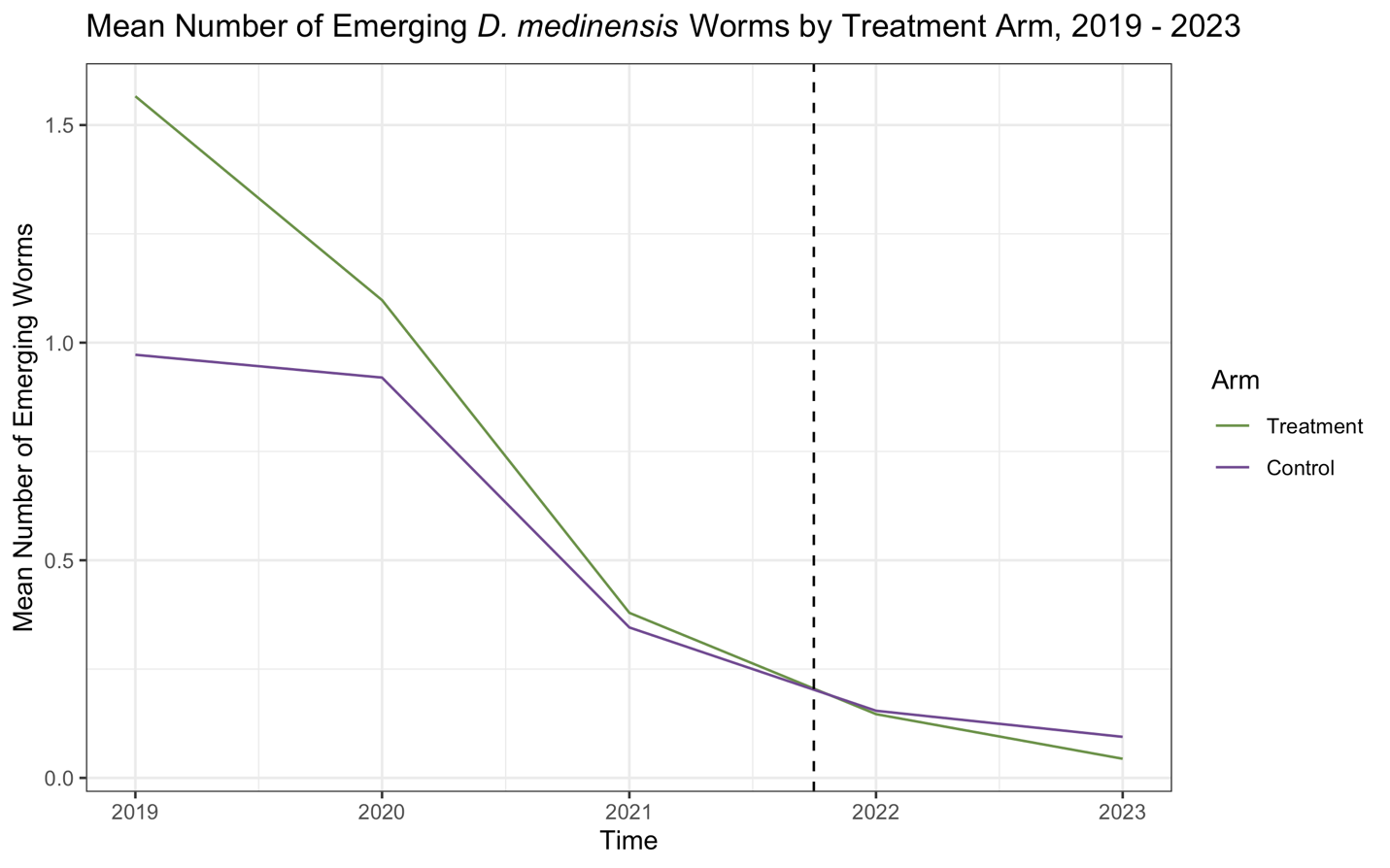

Supplement: S1 Fig — (DOCX) [file pntd.0014482.s001.docx]
