# Supplementary material for: Flubendazole 2.0: Designing a large field trial in dogs for a challenging setting
Source: PLoS Negl Trop Dis. 2026 Jul 2;20(7):e0014482. doi: 10.1371/journal.pntd.0014482 (PMC13345460; doi:10.1371/journal.pntd.0014482)
Supplement: Attachment — Submitted filename: Response to Reviewers.docx [file pntd.0014482.s003.docx]

March 2, 2026

Dear Reviewers,

Thank you for your critical and supportive comments. We have used your comments to make changes in the text, and we think that the article has improved as a result. We have responded to each comment in detail below and made the corresponding changes in the manuscript text.

Sincerely,

Amy Dupper

**Comments to the Authors:**

**Reviewer #1**

**Overview**

This manuscript addresses a critical and pressing issue in public health, i.e. Guinea worm disease (GWD) eradication and the challenges posed by the emergence of dogs as a primary reservoir. The authors outline the obstacles faced during the first clinical trial of flubendazole and propose a refined study design for the second trial. While the manuscript contributes to an important area of research including a smart design to tackle existing hurdles, there are several areas of improvement and additional expansion required for a comprehensive presentation.

**Overarching concerns**

1. One of the most important methodological aspects mentioned is the use of a Difference-in-Differences (DID) model to address challenges in analysing the trial outcomes. While this is a great approach given the conditions, the authors should expand their discussion on the pros and cons of using such a model. A DID model can control for time-invariant confounding factors, which is crucial in field trials with high levels of uncontrolled variability. It accounts for trends over time, enabling differentiation between the effects of the intervention and background noise. DID assumes parallel trends between intervention and control groups, which might be difficult to validate in such settings and is strongly based on expected effect sizes. This could be outlined in more detail. It is limited in handling time-varying confounding factors, which might play a role in field settings with seasonal or environmental changes. Expanding this section to include newer citations and examples of DID applications in similar public health or parasitological studies would strengthen the manuscript. Additionally, these pros and cons could be more clearly tied to the context of this study, emphasizing why DID is particularly suitable to mitigate confounding from environmental and study site variability.

Thank you for this comment! We have expanded on our reasoning for using a DID approach to analyzing these data from the clinical trial, including updated citations from methods papers and books about DID. Specifically, we included updated citations from studies that have used DID to analyze a policy or intervention on a neglected infectious disease. We also expounded on how we assessed the parallel trends assumption. [L246-L272]

2. The manuscript mentions that "the count of emerging worms per village" was used as a key parameter in the study design. However, it is not evident why this specific parameter was selected. It is important to thoroughly explain:
   - Why this metric is clinically relevant.

Thank you for this comment. If FLBZ reduces the fecundity of larvae, the parameter of lagged yearly count of emerging *D. medinensis* worms provides a more sensitive measure compared to the number of hosts with emerging *D. medinensis* worms because hosts can have more than one emerging *D. medinensis* each year. We have included the following rationale for using this parameter:

“Specifically, we chose to look at outcomes at the village level because population-level effects may be more appropriate given that FLBZ was shown to inhibit embryogenesis of L1s, resulting in reduced infectious larvae in local water bodies. Our outcome of interest was the yearly count of emerging *D. medinensis* female worms in subsequent years, which is a more sensitive measure of infectiousness present in a village compared to the number of hosts with emergent *D. medinensis* worms.” [L175-L181]

- What is the basis for the power calculation.

We used the number of emergent *D. medinensis* worms in our power calculation. A traditional sample size calculation was not available that would address the complexity of our research design. Therefore, we used existing data from active surveillance in Chad on the number of emerging *D. medinensis* worms per village to perform a power calculation to forecast how many villages are needed to detect a 50% reduction in emergent worms.

- State more clearly how this sample size differs from FBZ1.0 and how it impacts the power of the study.

The study designs for FLBZ 1.0 and FLBZ 2.0 were different. Specifically, FLBZ 1.0 assessed outcomes at the individual dog level (N = 435), whereas FLBZ 2.0 assessed outcomes at the village level (N = 56). The power analysis for FLBZ 2.0 is based on the number of villages needed to detect a 50% reduction in the outcome, so the number of individual dogs enrolled is less important to the outcome of interest. We have included the following sentence justifying our decision to assess outcomes at the village-level:

“Specifically, we chose to look at outcomes at the village level because population-level effects may be more appropriate given that FLBZ was shown to inhibit embryogenesis of L1s, resulting in reduced infectious larvae in local water bodies. Our outcome of interest was the yearly count of emerging *D. medinensis* female worms in subsequent years, which is a more sensitive measure of infectiousness present in a village compared to the number of hosts with emergent *D. medinensis* worms.” [L175-L181]

- How it captures the efficacy of flubendazole (FBZ 1.0), especially when emerging worms are captured, but no activity of FBZ on L3s are expected. It would be helpful to explain more clearly that at relevant discussion sections that if impacting the adult worm and embryogenesis, it would affect the number of emerging worms indirectly.

Thank you for this comment. We attempted to explain how we expected FLBZ to act on the developing L1s in the gravid female and how this mechanism of action would impact the transmissibility of *D. medinensis* to subsequent hosts. We have included the following explanation:

“That is female *D. medinensis* worms in dogs treated with FLBZ would still emerge and L1s would be released into the water, but these L1s would exhibit reduced ability to infect copepods. Therefore, mammalian hosts drinking from bodies of water would have a lower probability of consuming copepods with infectious L3s. Instead of observing a reduced incidence in FLBZ-treated dogs after 12 months compared to control dogs, we would expect to see a reduced incidence in all dogs regardless of treatment receipt in a FLBZ-treated village beginning 24 months after treatment start compared to dogs in control villages due to a reduction in transmission potential.” [L502-L509]

- Whether it was the only parameter used and how it complements other potential metrics (e.g., prevalence in dogs or humans, reduction in transmission rates). Providing additional details on these aspects would enhance clarity and contextualize the study’s analytical approach.

The count of emerging *D. medinensis* female worms was the only parameter used in the DID analyses. This parameter measures the yearly incidence of emerging worms, which is a more specific parameter compared to prevalence for this disease as hosts can have more than one worm each year. Transmission rate would be interesting to explore, but this measurement would require genetics or knowing the infectiousness of the water, which is not currently possible.

We collected all emergent *D. medinensis* worms from dogs during the study period and scored each gravid worm based on its reproductive morphology. The results from this analysis are included in the manuscript detailing the results from the FLBZ 2.0 trial. Prior work in lab ferrets during FLBZ 1.0 to assess motility of L1s from emerging female worms was not feasible in the field for FLBZ 2.0.

3. While the manuscript acknowledges challenges from the first trial, it would benefit from a direct comparison between the first trial’s outcomes and the improvements expected in the second trial with the modified study design. This would clarify the unique contribution of the authors’ work.

Thank you for this comment. We have included a more explicit comparison of the challenges encountered in FLBZ 1.0 and outlined how we designed FLBZ 2.0 to address these challenges. [L174-L181]

4. The manuscript states that the study was conducted between 2021 and 2022, but it is unclear why the design is being presented only in 2025. Given this timeline, it is expected that the results of the study would already be available. The absence of results is a significant gap that limits the completeness of the manuscript. The authors should:
   - Either include the results of the study in this manuscript.
   - Or justify the delay in publication and explicitly state when the results will follow.

Thank you for this comment! We agree that there is a significant time gap between when the trial finished and when this manuscript was submitted to the journal. Another manuscript was recently submitted to *PLoS Neglected Tropical Diseases* [PNTD-D-26-00062] where we present all the results from the trial. We had originally expected to publish the methods and results in the same manuscript but later decided to publish the methods of the trial and results separately, which allowed us to explain both in detail, whereas a single submission would have been prohibitively long. This delayed the writing of the methods and results manuscript as we were finalizing the surveillance numbers and results. Publication was further delayed after circulating the manuscript with the Carter Center (funders) and our collaborators in Chad for feedback. Their priorities with the eradication program and ongoing projects delayed our time to submission.

We have made it more explicit in the manuscript that the results are presented in a separate manuscript. [L43, L195-L196, L485-L486]

**Reviewer #2**

**Overview**

This manuscript describes the design of an experiment for the treatment of Guinea worm disease in dogs. Lessons learned from a preliminary trial informed the study design of the proposed trial, including follow-up length and the approach to allocating treatments within and between villages. The manuscript is interesting to read and the methods appear to be sound. However, several important definitions and clarifications are needed:

**Major comments:**

1. The primary outcome is not sufficiently defined. The authors state that “the outcome was the count of emerging worms per village” (line 224), and that “the average change in the outcome was estimated” (line 229). It must be clearly specified whether primary outcome is based on absolute counts or on differences/changes from baseline. Additionally, it should be defined which follow-up times are related to the primary endpoint. Are enrollment groups combined for the data analysis?

Thank you for this comment. We have included clarifying language where we state that “…our dependent variable was the yearly count of emerging *D. medinensis* female worms per village. The DID analysis specifically measures the average treatment effect on the treated, so the estimate reported from the analysis was the average yearly change in the count of emerging *D. medinensis* female worms in FLBZ-treated villages compared to control villages post-treatment compared to pre-treatment…” [L247-L251]

The dogs enrolled in FLBZ-treated villages were followed and multiple enrollments rounds were performed over the study period to ensure the assumptions of the analysis remained valid (non-differential attrition, consistent dose of treatment over time). The individual dog outcomes are not relevant to the main analysis. The primary endpoint relates to outcomes measured at one and two years after treatment start at the village level.

2. The endpoint is a raw count (or difference in counts) and it may be influenced by the total number of dogs in a village. How do the authors account for that, e.g., if dog numbers change? ? The referenced article [37] discusses relative emergence rates—are relative measures considered in the current study, and if not, why?

We changed this citation [37], and we did not use relative emergence rates. When designing the study, we looked at the influence of dog population on the count of emerging *D. medinensis* female worms but did not find an association. Because the dog census data is collected in a non-systematic way (see Table 1), we thought including total dog population in the model would introduce more bias. With the presence of a village-level random effect, the unknown dog population size is part of the random effect for that village.

3. The follow-up is not sufficiently defined in the Methods section. The authors state, that a one-year follow-up time may be insufficient, but they do not specify the actual follow-up of this study. A two-year follow-up is mentioned only in the Discussion, and it remains unclear whether all enrolment groups share the same follow-up schedule. This requires clarification.

Thank you for catching this omission. We have included dates when the study began and ended and dates for follow-ups as it relates to tracking individual dogs to record attrition. “The study began in October 2021 and ended in May 2023, and the seasonal trend of the disease informed the timing of the first FLBZ dose. Dogs were recruited into the study beginning in October 2021 right after peak emergence and when L3s would be present in dogs. Dogs were followed up approximately every three months in February 2022, May 2022, September 2022, November 2022, and May 2023 with additional recruitments occurring in May 2022 and November 2022 in order to maintain the population dose of FLBZ.” [L387-L392]

Regarding the data used for the DID analysis, we have included information on the timeframe for when yearly outcomes measured at the village level were included. “We compared yearly outcomes at the population level pre-treatment (November 2019 – October 2021) to population level yearly outcomes post-treatment (November 2021 – October 2023).” [L251-L253]

4. The authors performed a simulation study for the sample size calculation, the village number power analysis assumes a 50% decrease between treatment groups. The manuscript should provide a more detailed description of the simulations study, including the (distributional) assumptions, parameter choices etc.

Thank you for this comment. We have added additional clarifying language in the section describing the power analysis that includes distributional assumptions and parameter choices. Because the simulated outcome is a count of cases, we used a Poisson regression model. For the simulation, we assumed that, in the absence of the intervention, the rates in the post-intervention period (2022) would have been similar to those observed in previous years. Among the treated villages, we assumed a 50% reduction in the rate following the intervention. [L284-L305]

5. Line 292 mentions a join-count test which is not defined, the associated reference does not include the keyword. In line 295 this is called joint-count statistics. This paragraph lacks sufficient details and should be rewritten to clearly describe the test, its purpose, and its assumptions.

We have revised this section to include a brief description of the join-count test, its null hypothesis, and the other software used to conduct the analysis. [L339-L351]

6. The role of individual dog in the study design is not clear: While the methods section states, that enrolled dogs receive microchips to allow for consistent identification at each follow-up and to make sure that treated and control dogs were present (and to track them if they moved), the Discussion states, that it is not necessary to track individual dogs (line 446). This should be clarified.

Thank you for pointing out these conflicting statements. We attempted to enroll all dogs in each enrolled village, and we gave microchips to dogs to allow for identification at each follow-up. Because a significant challenge in FLBZ 1.0 was the high attrition, we wanted to track individual dogs to keep track of attrition over the study period. In several instances, dogs moved from one village to another during the study, so tracking these dogs’ movements was important in identifying non-differential attrition and population-level dose of treatment over time.

We have removed the line stating that it is not necessary to track individual dogs. We have included clarification that our outcome for the analysis was not dependent on individual dog data. [L495-L497]

7. The manuscript focuses on the methodological aspects of the clinical trial, the presentation of results thus is understandably short. However, important information about the current status of the trial is missing. The authors should describe whether the trial is still ongoing, completed, or terminated. If results from the trial have already been published elsewhere, this should be explicitly stated and the relevant references provided.

Thank you! We have tried to clearly state that the main results from the trial will be published separately. They are currently under review at *PLoS Neglected Tropical Diseases.* [L43, L195-L196, L485-L486]

**Minor comments:**

1. The abbreviation GWEP is not defined.

Thank you for catching this. We have added in the Guinea Worm Eradication Program with the first mention of this abbreviation. [L205]

2. Please critically review the following reference list to ensure that all citations are accurate and appropriately matched to the statements in the text:

Thank you for catching these citation errors! We have gone through and updated the citations to match the statements in the text.

a. Line: 147: Reference [34]

We have updated this citation to [35].

b. Line 225: Reference [37];

This citation was removed.

c. line 292: Reference [41]

This citation is now correct.

**Reviewer #3**

**Overview**

This paper presents an enhanced study protocol for controlling Guinea worm (D. medinensis) in peri-domestic dogs in Chad. Dogs play a significant role in parasite transmission, and reducing infections within this animal reservoir is a critical step towards eradication. The study outlines how flubendazole treatment will be implemented to detect differences in emerging infections between treated and untreated villages. The study carefully addresses design challenges and provides valuable insights for similar field trials and highlights the challenges of conducting large-scale randomized clinical trials in resource-limited settings.

**Major comments:**

1. Seasonality and study design considerations
   a. Line 113: The authors state that Guinea worm infections in dogs follow a seasonal pattern, most frequently occurring between March and September. Is there a biological or ecological explanation for this seasonality (e.g. association with the rainy season, water availability, or seasonal fishing practices)? Was this seasonal trend considered in the study design (e.g. enrollment, timing of treatment)?

The seasonality of Guinea worm infections has both an ecological and biological explanation. Peak transmission aligns with the end of the hot/dry season and the beginning of the rainy season when water levels are at their lowest [L118-L120]. This also coincides with when fishing activities are most prevalent, and the exposure to this transmission pathway is greatest for dogs in Chad. Additionally, the prepatent period, or time from exposure to infectious L3s to when the gravid female worm emerges from the host, is one year, which also facilitates the yearly seasonal pattern observed.

The first enrollment wave was done in October 2021 towards the beginning of the dry season and following peak transmission of the parasite. This is when L3s were assumed to be in dogs. The drug was assumed to slowly release into the tissues over six months, such that exposure to the drug was consistent over time. The second dose of treatment was done in May 2022, when the treatment would be due. [L387-L389]

2. Baseline incidence and prior intervention phase
   a. Line 143: Please report the average Guinea worm incidence during the FLBZ 1.0 phase. Given the partial geographic overlap with the current study area, this information is important for considering baseline transmission and expected treatment effects. Additionally, it would be helpful to include information on when the study was conducted.

Thank you for this comment. We have included mean percent prevalence of GW infections in dogs during FLBZ 1.0 because this is the parameter we have. The prior incidence in these villages as well as others in the region for 2019-2020 was considered in the sample size calculation. [L151-L153]

3. Village comparability and potential confounding factors
   a. Line 282: Sixty villages were included in the study. Please clarify whether villages were comparable with respect to key parameters such as geographic location, access to water sources, number of dogs, and environmental exposure risk.

We feel as though we addressed comparability of study villages in both the sections on inclusion and exclusion criteria.

b. Line 187-190: Although town and suburb sites are uniformly referred as villages, a summary table describing basic characteristics (e.g. human population size, dog population size, access to water, level of development, proximity to aquatic environments) would improve transparency. Differences in these factors could influence reinfection rates, transmission intensity, treatment effects and should also be considered in the analysis.

While these characteristics exist, we do not have access to them. Table 1 gives an idea of the size of village by showing the census of dog populations in each enrolled village, and Figure 1 shows that all enrolled villages are located close to the Chari River. Reinfection rates could have to do with availability of safe drinking sources (i.e., pumps), this is more applicable for human infections. Proactive tethering of dogs is more indicative of reinfection, and we only enrolled villages that were enrolled in the proactive tethering program.

4. Surveillance, diagnosis, and incidence estimation
   a. Surveillance methods: Please describe in detail how surveillance for Guinea worm infections was conducted during the study period. Was detection based on visual confirmation of emerging worms by owners or team members? Were blister formation or skin lesions considered, and was any laboratory confirmation (e.g. PCR) performed?

Thank you for this comment. We have included additional details about how surveillance activities identified GW infections.

“PNEVG-T only recorded *D. medinensis* infections if the worm was actively emerging from the host; blisters and lesions were not recorded as infections. Upon emergence, the worm was visually identified by PNEVG-T staff, and a piece was sent to CDC and Vassar College where microscopic identification and sequencing were, respectively, performed to verify the worm was *D. medinensis.*” [L411-L415]

b. Diagnostic criteria for inclusion: Please clarify the diagnostic criteria used for study inclusion. If diagnosis relied primarily on the presence of an emerging adult worm, early infections may have been missed. What measures were taken to minimize underestimation of true incidence? This is important, as the evaluation of FLBZ efficacy relies on infection counts.

This is a good point! Male worms, early infections, and worms that never emerge may have been missed. While the count of emerging *D. medinensis* is not the most sensitive measure, it was the most feasible for field research. We collected serum and plasma from dogs at each follow-up to be used in emerging diagnostics, but none have been sufficiently validated as of the writing of this manuscript to use their results as outcomes for comparison. We hope to use these in the future as a more sensitive method to detect infection regardless of worm emergence.

We have included a sentence in the discussion section acknowledging that this parameter is not a perfect measure of true incidence. [L574-L576]

5. Exposure routes and exclusion criteria
   a. Line 215-219: Some villages were excluded to reduce the likelihood of including dogs infected through fish consumption rather than contaminated water sources. How was the infection route determined for included dogs? How can fish-borne transmission be excluded in the remaining villages, and how might misclassification affect study conclusions?

The designation of some villages as “fishing villages” was a designation given by the Carter Center. However, we were not able to ascertain the transmission route of the dogs in this study. We attempted to mitigate the bias introduced by enrolling villages with dogs with higher exposure to a fish-borne route of transmission by excluding fishing villages, but, of course, it is always possible that bias still exists.

As Fig 1 shows, we enrolled villages to the treatment and control arms that were all located near the Chari River, and thus had access to similar bodies of water. Additionally, using a DID analysis assists in mitigating any bias introduced by misclassification because we are comparing each village post-treatment to itself pre-treatment. Background transmission of GW by fish from outside of a village, if present, would likely bias the results towards the null.

6. Treatment exclusions and transmission implications
   a. Line 309: Pregnant dogs were excluded from treatment. Were pregnancy rates compared across villages and between treatment arms to ensure comparability? As untreated pregnant dogs may continue to act as a reservoir for transmission, differences in pregnancy rates could confound treatment effects and should be considered.

Thank you for this comment! We do not have consistent data on the number of pregnant dogs that were excluded in October 2021. We looked at the number of female dogs enrolled in May 2022 (which should include dogs excluded during the first enrollment round), and there was no difference in the mean number of new female dogs enrolled in the FLBZ and control arms (312 vs 226 dogs, p = 0.45). [L363-L366]

We know that excluding female dogs would bias the effect of treatment towards the null, so we trust that an effect of treatment would be an underestimation.

7. Follow-up procedures and outcome assessment
   a. Line 343-344: The total number and timing of follow-up visits are not entirely clear. Please clarify whether follow-ups were conducted every three months for up to two years post-treatment.

Thank you for pointing out this omission. We have included language clarifying that follow-ups were approximately every six months (October 2021, February 2022, May 2022, September 2022, November 2022, and May 2023) with dog enrollments occurring in October 2021, May 2022, and November 2022. The follow-ups and reenrollments were to track attrition of dogs and maintain a population “dose” of FLBZ. The main DID analysis did not rely on individual dog data but instead compared the yearly number of emerging *D. medinensis* per village in treated villages compared to control villages and comparing the difference pre-treatment (November 2019 – October 2021) to post-treatment (November 2021 – October 2023). The data used in the DID analysis came from active surveillance that is collected continuously by PNEVG-T. [L387-L392]

b. Line 345: During follow-up examinations, emerging subcutaneous worms were documented. Were additional parameters recorded, such as the number of infection sites, number or length of adult worms, or indicators of infection intensity? Was wound exposure to water performed to quantify released larvae, or were larval motility scores assessed? These measures could strengthen evaluation of treatment efficacy.

These parameters were not recorded as part of the follow-up. Any wound that looked like it could possibly an emerging *D. medinensis* was recorded, and surveillance teams were alerted. All identified emerging worms were extracted by PNEVG-T, but this was not a typical activity performed by their staff. Blood and serum of all enrolled dogs were collected at each follow-up, but analyses on these samples have not yet been done. This would give a better idea of the efficacy of the treatment, but the aim of this study was not to assess efficacy but rather the effect of the drug on annual incidence of GWD. [L412-L415]

8. Mortality and competing risks
   a. Line 416 and 425: The reported mortality rate of 27–34% in enrollment groups 1–3 is high. While a canine distemper virus outbreak is given as potential reason for dogs enrolled in November 2022, this does not fully explain the consistently elevated mortality across all time points. Were other causes of death (e.g. age, accidents, malnutrition, rabies) assessed or recorded? Please clarify how mortality was accounted for in the analysis.

Thank you for this comment. Yes, we recorded owner-reported causes of death for all dogs in the study. In general, the causes of death were unknown or vague. Our observed mortality is consistent with what is observed in other studies of dogs in similar settings. This was one of the motivating reasons for the village-level analysis. As long as the mortality was similar in the two arms and not associated with the treatment or outcome, then high mortality would not bias the analysis or reduce the power.

9. Evaluation of treatment efficacy
   a. Line 435: Please describe in detail how the impact of FLBZ treatment on Guinea worm infections was evaluated. Was efficacy assessed based on infection counts per dog, reinfections, number of infection sites, adult worm burden or length, or characteristics of released larvae (e.g. motility)? A clearer definition of outcome measures would strengthen interpretation.

We have changed the wording to clarify that we were measuring the effect of treatment by examining the number of emerging *D. medinensis* gravid female worms among domestic Chadian dogs in each village. In other words, we used infection counts in dogs per village as a measure of overall disease burden in each village. [L175-L181]

10. Pharmacokinetic assumptions
    a. Line 339: At a concentration of 100 mg/kg flubendazole, a slow release over approximately six months is assumed. Were pharmacokinetic measurements (e.g. blood sampling during follow-up to assess FLBZ concentrations or half-life) considered or planned?

Thank you for this comment. A prior pharmacokinetic study was performed (results not published yet), which failed to find consistent levels of the drug in plasma collected from treated dogs. The results of that study were not available until near the end of the FLBZ 2.0 field study. Some dogs in the PK study had spikes of the drug in their serum while others had no detectable levels. No pharmacokinetic study was done on the serum or subcutaneous tissues from dogs in this study, which limits the inferences we can make about the effects of FLBZ on emerging *D. medinensis*.

11. Environmental transmission context
    a. Line 500: Given the importance of aquatic environments in Guinea worm transmission, the authors may consider whether the protocol could be expanded to include environmental measurements, such as sampling water bodies for larvae, assessing copepod density, or estimating the proportion of infected copepods.

This is an excellent suggestion, and while this study did not collect environmental samples, our colleagues are preparing a manuscript on this topic. They collected water samples, some from villages included in this study, and counted the copepod density and tested for *D. medinensis* DNA.

12. Human infections and One Health perspective
    a. Do human Guinea worm infections still occur in the study area? If so, were these monitored or recorded during the study period, and were they considered in the study protocol or intervention strategy?

Thank you for this comment. Human GW cases are rare and occur sporadically (only 6 cases in 2022 and 9 cases in 2023), some of which occurred in the study area over the study period. This study focused on dogs as they are the main definitive host in the country (and globally, at the time of the study). The Chadian Guinea Worm Eradication Program has ongoing intensive surveillance and interventions for humans with GWD such that it can reasonably be assumed that human cases do not transmit the parasite on to others, so this was not considered in the study protocol.

13. Rabies vaccination context
    a. Since all enrolled dogs reportedly received rabies vaccination, is rabies endemic in the study area? If available, please provide information on rabies vaccine coverage among enrolled dogs and whether vaccination status differed between villages or study arms.

Rabies is endemic in the study area, and providing rabies vaccines was offered as an incentive for participating as well as a public health and occupational health measure. All dogs enrolled in the study, whether in the treatment or control arm, were administered a rabies vaccine. Very few dogs in the area received rabies vaccine at the time of the study as noted in this paper doi: [10.3389/fvets.2022.866755](https://doi.org/10.3389/fvets.2022.866755). Subsequent to the study, the proactive tethering program began vaccinating tethered dogs.

**Minor comments:**

1. Figure 2: For the figure showing attrition of enrolled dogs over the study period, the authors may consider splitting alive dogs into treatment and control groups to improve clarity and interpretability. This suggestion is optional and can be disregarded if it does not lead to a clearer presentation or is potentially confusing.

Thank you for the suggestion. We have examined these figures in many different ways and the version that we chose was the most clear and informative in our opinion.
