# Supplementary material for: Flubendazole 2.0: Designing a large field trial in dogs for a challenging setting
Source: PLoS Negl Trop Dis. 2026 Jul 2;20(7):e0014482. doi: 10.1371/journal.pntd.0014482 (PMC13345460; doi:10.1371/journal.pntd.0014482)
Supplement: Peer review history

# Peer review: Decision Letter 0

**DOI:** 10.1371/journal.pntd.0014482.r001

**Authors:** Krystyna Cwiklinski, Marc Hübner

5 Jan 2026

-->PNTD-D-25-01525

Flubendazole 2.0: designing a large field trial in dogs for a challenging setting

PLOS Neglected Tropical Diseases

Dear Dr. Dupper,

Thank you for submitting your manuscript to PLOS Neglected Tropical Diseases. After careful consideration, we feel that it has merit but does not fully meet PLOS Neglected Tropical Diseases's publication criteria as it currently stands. Therefore, we invite you to submit a revised version of the manuscript that addresses the points raised during the review process.

Please submit your revised manuscript within by Mar 06 2026 11:59PM. If you will need more time than this to complete your revisions, please reply to this message or contact the journal office at plosntds@plos.org. When you're ready to submit your revision, log on to https://www.editorialmanager.com/pntd/ and select the 'Submissions Needing Revision' folder to locate your manuscript file.

Please include the following items when submitting your revised manuscript:

* A letter that responds to each point raised by the editor and reviewer(s). You should upload this letter as a separate file labeled 'Response to Reviewers'. This file does not need to include responses to any formatting updates and technical items listed in the 'Journal Requirements' section below.

* A marked-up copy of your manuscript that highlights changes made to the original version. You should upload this as a separate file labeled 'Revised Manuscript with Track Changes'.

* An unmarked version of your revised paper without tracked changes. You should upload this as a separate file labeled 'Manuscript'.

If you would like to make changes to your financial disclosure, competing interests statement, or data availability statement, please make these updates within the submission form at the time of resubmission. Guidelines for resubmitting your figure files are available below the reviewer comments at the end of this letter.

We look forward to receiving your revised manuscript.

Kind regards,

Marc P Hübner

Academic Editor

PLOS Neglected Tropical Diseases

Krystyna Cwiklinski

Section Editor

PLOS Neglected Tropical Diseases

Shaden Kamhawi

co-Editor-in-Chief

PLOS Neglected Tropical Diseases

orcid.org/0000-0003-4304-636XX

Paul Brindley

co-Editor-in-Chief

PLOS Neglected Tropical Diseases

orcid.org/0000-0003-1765-0002

**Additional Editor Comments:**

The manuscript presents a revised study design to overcome challenges that were observed in the initial flubendazole trial on dogs with Guinea worm. The reviewers acknowledged the importance of this study, but identified several points that have to be addressed by the authors. This includes, among others, the definition and justification of the primary outcomes and endpoints, additional methodological clarifications and the status of the study. The absence of results significantly limits the manuscript’s completeness. Please either include study outcomes or clearly justify the presentation of a design-only manuscript and specify when results will be reported.

**Journal Requirements:**

If the reviewer comments include a recommendation to cite specific previously published works, please review and evaluate these publications to determine whether they are relevant and should be cited. There is no requirement to cite these works unless the editor has indicated otherwise.

1) Some material included in your submission may be copyrighted. According to PLOSu2019s copyright policy, authors who use figures or other material (e.g., graphics, clipart, maps) from another author or copyright holder must demonstrate or obtain permission to publish this material under the Creative Commons Attribution 4.0 International (CC BY 4.0) License used by PLOS journals. Please closely review the details of PLOSu2019s copyright requirements here: PLOS Licenses and Copyright. If you need to request permissions from a copyright holder, you may use PLOS's Copyright Content Permission form.

Please respond directly to this email and provide any known details concerning your material's license terms and permissions required for reuse, even if you have not yet obtained copyright permissions or are unsure of your material's copyright compatibility. Once you have responded and addressed all other outstanding technical requirements, you may resubmit your manuscript within Editorial Manager.

Potential Copyright Issues:

i) Figure 1. Please (a) provide a direct link to the base layer of the map (i.e., the country or region border shape) and ensure this is also included in the figure legend; and (b) provide a link to the terms of use / license information for the base layer image or shapefile. We cannot publish proprietary or copyrighted maps (e.g. Google Maps, Mapquest) and the terms of use for your map base layer must be compatible with our CC BY 4.0 license.

Note: if you created the map in a software program like R or ArcGIS, please locate and indicate the source of the basemap shapefile onto which data has been plotted.

If your map was obtained from a copyrighted source please amend the figure so that the base map used is from an openly available source. Alternatively, please provide explicit written permission from the copyright holder granting you the right to publish the material under our CC BY 4.0 license.

If you are unsure whether you can use a map or not, please do reach out and we will be able to help you. The following websites are good examples of where you can source open access or public domain maps:

* U.S. Geological Survey (USGS) - All maps are in the public domain. (http://www.usgs.gov)

* PlaniGlobe - All maps are published under a Creative Commons license so please cite u201cPlaniGlobe, http://www.planiglobe.com, CC BY 2.0u201d in the image credit after the caption. (http://www.planiglobe.com/?lang=enl)

* Natural Earth - All maps are public domain. (http://www.naturalearthdata.com/about/terms-of-use/).

2) Since your data is not available for proprietary reasons, please explain via email why the data is not available. Please also include the contact information for the third party organization that should be contacted should other researchers want to request access to this data and please include the full citation of where the data can be found. We also request that you verify with us via email that any researcher will be able to obtain the data set in the same manner that the you have obtained it.

If you feel you are unwilling or unable to adhere to this policy, please explain your reasons by return email and your exemption request will be escalated to the editor for approval. Your exemption request will be handled independently and will not hold up the peer review process, but will need to be resolved should your manuscript be accepted for publication. One of the Editorial team will be in touch if they require more information.

**Reviewers' Comments:**

Reviewer's Responses to Questions

**Key Review Criteria Required for Acceptance?**

As you describe the new analyses required for acceptance, please consider the following:

**Methods**

-Are the objectives of the study clearly articulated with a clear testable hypothesis stated?

-Is the study design appropriate to address the stated objectives?

-Is the population clearly described and appropriate for the hypothesis being tested?

-Is the sample size sufficient to ensure adequate power to address the hypothesis being tested?

-Were correct statistical analysis used to support conclusions?

-Are there concerns about ethical or regulatory requirements being met?

Reviewer #1: See below

Reviewer #2: This manuscript describes the design of an experiment for the treatment of Guinea worm disease in dogs. Lessons learned from a preliminary trial informed the study design of the proposed trial, including follow-up length and the approach to allocating treatments within and between villages. The manuscript is interesting to read and the methods appear to be sound. However, several important definitions and clarifications are needed:

The primary outcome is not sufficiently defined. The authors state that “the outcome was the count of emerging worms per village” (line 224), and that “the average change in the outcome was estimated” (line 229). It must be clearly specified whether primary outcome is based on absolute counts or on differences/changes from baseline. Additionally, it should be defined which follow-up times are related to the primary endpoint. Are enrollment groups combined for the data analysis?

The endpoint is a raw count (or difference in counts) and it may be influenced by the total number of dogs in a village. How do the authors account for that, e.g., if dog numbers change? ? The referenced article [37] discusses relative emergence rates—are relative measures considered in the current study, and if not, why?

The follow-up is not sufficiently defined in the Methods section. The authors state, that a one-year follow-up time may be insufficient, but they do not specify the actual follow-up of this study. A two-year follow-up is mentioned only in the Discussion, and it remains unclear whether all enrolment groups share the same follow-up schedule. This requires clarification.

The authors performed a simulation study for the sample size calculation, the village number power analysis assumes a 50% decrease between treatment groups. The manuscript should provide a more detailed description of the simulations study, including the (distributional) assumptions, parameter choices etc.

The abbreviation GWEP is not defined.

Line 292 mentions a join-count test which is not defined, the associated reference does not include the keyword. In line 295 this is called joint-count statistics. This paragraph lacks sufficient details and should be rewritten to clearly describe the test, its purpose, and its assumptions.

The role of individual dog in the study design is not clear: While the methods section states, that enrolled dogs receive microchips to allow for consistent identification at each follow-up and to make sure that treated and control dogs were present (and to track them if they moved), the Discussion states, that it is not necessary to track individual dogs (line 446). This should be clarified.

Reviewer #3: 1. Seasonality and study design considerations

Line 113: The authors state that Guinea worm infections in dogs follow a seasonal pattern, most frequently occurring between March and September. Is there a biological or ecological explanation for this seasonality (e.g. association with the rainy season, water availability, or seasonal fishing practices)? Was this seasonal trend considered in the study design (e.g. enrollment, timing of treatment)?

2. Baseline incidence and prior intervention phase

Line 143: Please report the average Guinea worm incidence during the FLBZ 1.0 phase. Given the partial geographic overlap with the current study area, this information is important for considering baseline transmission and expected treatment effects. Additionally, it would be helpful to include information on when the study was conducted.

3. Village comparability and potential confounding factors

Line 282: Sixty villages were included in the study. Please clarify whether villages were comparable with respect to key parameters such as geographic location, access to water sources, number of dogs, and environmental exposure risk.

Line 187-190: Although town and suburb sites are uniformly referred as villages, a summary table describing basic characteristics (e.g. human population size, dog population size, access to water, level of development, proximity to aquatic environments) would improve transparency.

Differences in these factors could influence reinfection rates, transmission intensity, treatment effects and should also be considered in the analysis.

4. Surveillance, diagnosis, and incidence estimation

Surveillance methods: Please describe in detail how surveillance for Guinea worm infections was conducted during the study period. Was detection based on visual confirmation of emerging worms by owners or team members? Were blister formation or skin lesions considered, and was any laboratory confirmation (e.g. PCR) performed?

Diagnostic criteria for inclusion: Please clarify the diagnostic criteria used for study inclusion. If diagnosis relied primarily on the presence of an emerging adult worm, early infections may have been missed. What measures were taken to minimize underestimation of true incidence? This is important, as the evaluation of FLBZ efficacy relies on infection counts.

5. Exposure routes and exclusion criteria

Line 215-219: Some villages were excluded to reduce the likelihood of including dogs infected through fish consumption rather than contaminated water sources. How was the infection route determined for included dogs? How can fish-borne transmission be excluded in the remaining villages, and how might misclassification affect study conclusions?

6. Treatment exclusions and transmission implications

Line 309: Pregnant dogs were excluded from treatment. Were pregnancy rates compared across villages and between treatment arms to ensure comparability? As untreated pregnant dogs may continue to act as a reservoir for transmission, differences in pregnancy rates could confound treatment effects and should be considered.

7. Follow-up procedures and outcome assessment

Line 343-344: The total number and timing of follow-up visits are not entirely clear. Please clarify whether follow-ups were conducted every three months for up to two years post-treatment.

Line 345: During follow-up examinations, emerging subcutaneous worms were documented. Were additional parameters recorded, such as the number of infection sites, number or length of adult worms, or indicators of infection intensity? Was wound exposure to water performed to quantify released larvae, or were larval motility scores assessed? These measures could strengthen evaluation of treatment efficacy.

Additional points for consideration:

1. Pharmacokinetic assumptions

Line 339: At a concentration of 100 mg/kg flubendazole, a slow release over approximately six months is assumed. Were pharmacokinetic measurements (e.g. blood sampling during follow-up to assess FLBZ concentrations or half-life) considered or planned?

2. Environmental transmission context

Line 500: Given the importance of aquatic environments in Guinea worm transmission, the authors may consider whether the protocol could be expanded to include environmental measurements, such as sampling water bodies for larvae, assessing copepod density, or estimating the proportion of infected copepods.

3. Human infections and One Health perspective

Do human Guinea worm infections still occur in the study area? If so, were these monitored or recorded during the study period, and were they considered in the study protocol or intervention strategy?

4. Rabies vaccination context

Since all enrolled dogs reportedly received rabies vaccination, is rabies endemic in the study area? If available, please provide information on rabies vaccine coverage among enrolled dogs and whether vaccination status differed between villages or study arms.

**********

**Results**

-Does the analysis presented match the analysis plan?

-Are the results clearly and completely presented?

-Are the figures (Tables, Images) of sufficient quality for clarity?

Reviewer #1: See below

Reviewer #2: The manuscript focuses on the methodological aspects of the clinical trial, the presentation of results thus is understandably short. However, important information about the current status of the trial is missing. The authors should describe whether the trial is still ongoing, completed, or terminated. If results from the trial have already been published elsewhere, this should be explicitly stated and the relevant references provided.

Reviewer #3: Mortality and competing risks

Line 416 and 425: The reported mortality rate of 27–34% in enrollment groups 1–3 is high. While a canine distemper virus outbreak is given as potential reason for dogs enrolled in November 2022, this does not fully explain the consistently elevated mortality across all time points. Were other causes of death (e.g. age, accidents, malnutrition, rabies) assessed or recorded? Please clarify how mortality was accounted for in the analysis.

**********

**Conclusions**

-Are the conclusions supported by the data presented?

-Are the limitations of analysis clearly described?

-Do the authors discuss how these data can be helpful to advance our understanding of the topic under study?

-Is public health relevance addressed?

Reviewer #1: See below

Reviewer #2: No comments

Reviewer #3: Evaluation of treatment efficacy

Line 435: Please describe in detail how the impact of FLBZ treatment on Guinea worm infections was evaluated. Was efficacy assessed based on infection counts per dog, reinfections, number of infection sites, adult worm burden or length, or characteristics of released larvae (e.g. motility)? A clearer definition of outcome measures would strengthen interpretation.

**********

**Editorial and Data Presentation Modifications?**

Use this section for editorial suggestions as well as relatively minor modifications of existing data that would enhance clarity. If the only modifications needed are minor and/or editorial, you may wish to recommend “Minor Revision” or “Accept”.

Reviewer #1: (No Response)

Reviewer #2: Please critically review the following reference list to ensure that all citations are accurate and appropriately matched to the statements in the text:

Line: 147: Reference [34]

Line 225: Reference [37];

line 292: Reference [41]

Reviewer #3: Figure 2: For the figure showing attrition of enrolled dogs over the study period, the authors may consider splitting alive dogs into treatment and control groups to improve clarity and interpretability. This suggestion is optional and can be disregarded if it does not lead to a clearer presentation or is potentially confusing.

**********

**Summary and General Comments**

Use this section to provide overall comments, discuss strengths/weaknesses of the study, novelty, significance, general execution and scholarship. You may also include additional comments for the author, including concerns about dual publication, research ethics, or publication ethics. If requesting major revision, please articulate the new experiments that are needed.

Reviewer #1: This manuscript addresses a critical and pressing issue in public health, i.e. Guinea worm disease (GWD) eradication and the challenges posed by the emergence of dogs as a primary reservoir. The authors outline the obstacles faced during the first clinical trial of flubendazole and propose a refined study design for the second trial.

While the manuscript contributes to an important area of research including a smart design to tackle existing hurdles, there are several areas of improvement and additional expansion required for a comprehensive presentation.

1) One of the most important methodological aspects mentioned is the use of a Difference-in-Differences (DID) model to address challenges in analysing the trial outcomes. While this is a great approach given the conditions, the authors should expand their discussion on the pros and cons of using such a model. A DID model can control for time-invariant confounding factors, which is crucial in field trials with high levels of uncontrolled variability. It accounts for trends over time, enabling differentiation between the effects of the intervention and background noise. DID assumes parallel trends between intervention and control groups, which might be difficult to validate in such settings and is strongly based on expected effect sizes. This could be outlined in more detail. It is limited in handling time-varying confounding factors, which might play a role in field settings with seasonal or environmental changes. Expanding this section to include newer citations and examples of DID applications in similar public health or parasitological studies would strengthen the manuscript. Additionally, these pros and cons could be more clearly tied to the context of this study, emphasizing why DID is particularly suitable to mitigate confounding from environmental and study site variability.

2) The manuscript mentions that "the count of emerging worms per village" was used as a key parameter in the study design. However, it is not evident why this specific parameter was selected. It is important to thoroughly explain:

- Why this metric is clinically relevant.

- What is the basis for the power calculation.

- State more clearly how this sample size differs from FBZ1.0 and how it impacts the power of the study.

- How it captures the efficacy of flubendazole (FBZ 1.0), especially when emerging worms are captured, but no activity of FBZ on L3s are expected. It would be helpful to explain more clearly that at relevant discussion sections that if impacting the adult worm and embryogenesis, it would affect the number of emerging worms indirectly.

- Whether it was the only parameter used and how it complements other potential metrics (e.g., prevalence in dogs or humans, reduction in transmission rates). Providing additional details on these aspects would enhance clarity and contextualize the study’s analytical approach.

3) While the manuscript acknowledges challenges from the first trial, it would benefit from a direct comparison between the first trial’s outcomes and the improvements expected in the second trial with the modified study design. This would clarify the unique contribution of the authors’ work.

4) The manuscript states that the study was conducted between 2021 and 2022, but it is unclear why the design is being presented only in 2025. Given this timeline, it is expected that the results of the study would already be available. The absence of results is a significant gap that limits the completeness of the manuscript. The authors should:

- Either include the results of the study in this manuscript.

- Or justify the delay in publication and explicitly state when the results will follow.

Reviewer #2: No comments

Reviewer #3: This paper presents an enhanced study protocol for controlling Guinea worm (D. medinensis) in peri-domestic dogs in Chad. Dogs play a significant role in parasite transmission, and reducing infections within this animal reservoir is a critical step towards eradication. The study outlines how flubendazole treatment will be implemented to detect differences in emerging infections between treated and untreated villages. The study carefully addresses design challenges and provides valuable insights for similar field trials and highlights the challenges of conducting large-scale randomized clinical trials in resource-limited settings.

**********

PLOS authors have the option to publish the peer review history of their article (what does this mean?). If published, this will include your full peer review and any attached files.

If you choose “no”, your identity will remain anonymous but your review may still be made public.

**Do you want your identity to be public for this peer review?**  For information about this choice, including consent withdrawal, please see our Privacy Policy.

Reviewer #1: No

Reviewer #2: No

Reviewer #3: **Yes:**  Julia Meyer

**Figure resubmission:**

While revising your submission, we strongly recommend that you use PLOS’s NAAS tool (https://ngplosjournals.pagemajik.ai/artanalysis) to test your figure files. NAAS can convert your figure files to the TIFF file type and meet basic requirements (such as print size, resolution), or provide you with a report on issues that do not meet our requirements and that NAAS cannot fix.

-->After uploading your figures to PLOS’s NAAS tool - https://ngplosjournals.pagemajik.ai/artanalysis, NAAS will process the files provided and display the results in the "Uploaded Files" section of the page as the processing is complete. If the uploaded figures meet our requirements (or NAAS is able to fix the files to meet our requirements), the figure will be marked as "fixed" above. If NAAS is unable to fix the files, a red "failed" label will appear above. When NAAS has confirmed that the figure files meet our requirements, please download the file via the download option, and include these NAAS processed figure files when submitting your revised manuscript.-->

**Reproducibility:**

To enhance the reproducibility of your results, we recommend that authors of applicable studies deposit laboratory protocols in protocols.io, where a protocol can be assigned its own identifier (DOI) such that it can be cited independently in the future. Additionally, PLOS ONE offers an option to publish peer-reviewed clinical study protocols. Read more information on sharing protocols at https://plos.org/protocols?utm_medium=editorial-email&utm_source=authorletters&utm_campaign=protocols

-->

# Author comment: Author response to Decision Letter 1

**DOI:** 10.1371/journal.pntd.0014482.r002

15 May 2026

Attachment Submitted filename: Response to Reviewers.docx

# Peer review: Decision Letter 1

**DOI:** 10.1371/journal.pntd.0014482.r003

**Authors:** Krystyna Cwiklinski, Marc Hübner, Krystyna Cwiklinski, Marc Hübner

18 Jun 2026

Dear Ms. Dupper,

We are pleased to inform you that your manuscript 'Flubendazole 2.0: designing a large field trial in dogs for a challenging setting' has been provisionally accepted for publication in PLOS Neglected Tropical Diseases.

Before your manuscript can be formally accepted you will need to complete some formatting changes, which you will receive in a follow up email. A member of our team will be in touch with a set of requests.

Please note that your manuscript will not be scheduled for publication until you have made the required changes, so a swift response is appreciated.

IMPORTANT: The editorial review process is now complete. PLOS will only permit corrections to spelling, formatting or significant scientific errors from this point onwards. Requests for major changes, or any which affect the scientific understanding of your work, will cause delays to the publication date of your manuscript.

Should you, your institution's press office or the journal office choose to press release your paper, you will automatically be opted out of early publication. We ask that you notify us now if you or your institution is planning to press release the article. All press must be co-ordinated with PLOS.

Thank you again for supporting Open Access publishing; we are looking forward to publishing your work in PLOS Neglected Tropical Diseases.

Best regards,

Marc P Hübner, Prof. Dr.

Academic Editor

PLOS Neglected Tropical Diseases

Krystyna Cwiklinski

Section Editor

PLOS Neglected Tropical Diseases

Shaden Kamhawi

co-Editor-in-Chief

PLOS Neglected Tropical Diseases

orcid.org/0000-0003-4304-636XX

Paul Brindley

co-Editor-in-Chief

PLOS Neglected Tropical Diseases

orcid.org/0000-0003-1765-0002

***********************************************************

p.p1 {margin: 0.0px 0.0px 0.0px 0.0px; line-height: 16.0px; font: 14.0px Arial; color: #323333; -webkit-text-stroke: #323333}span.s1 {font-kerning: none

Reviewer's Responses to Questions

**Key Review Criteria Required for Acceptance?**

As you describe the new analyses required for acceptance, please consider the following:

**Methods**

-Are the objectives of the study clearly articulated with a clear testable hypothesis stated?

-Is the study design appropriate to address the stated objectives?

-Is the population clearly described and appropriate for the hypothesis being tested?

-Is the sample size sufficient to ensure adequate power to address the hypothesis being tested?

-Were correct statistical analysis used to support conclusions?

-Are there concerns about ethical or regulatory requirements being met?

Reviewer #1: (No Response)

Reviewer #2: -

Reviewer #3: The study objectives are clearly stated and well defined and the research question is scientifically relevant. The study design is appropriate and well suited to address the stated objectives. The methodological approach is clearly described, and allows the authors to adequately investigate the proposed research question. The study population and relevant characteristics are clearly described and the sample size appears sufficient to provide adequate statistical power. There are no apparent concerns regarding ethical or regulatory requirements.

**********

**Results**

-Does the analysis presented match the analysis plan?

-Are the results clearly and completely presented?

-Are the figures (Tables, Images) of sufficient quality for clarity?

Reviewer #1: (No Response)

Reviewer #2: -

Reviewer #3: The figures and tables are well organized, comprehensively presented and contain all relevant information which is necessary to understand the findings of the study.

**********

**Conclusions**

-Are the conclusions supported by the data presented?

-Are the limitations of analysis clearly described?

-Do the authors discuss how these data can be helpful to advance our understanding of the topic under study?

-Is public health relevance addressed?

Reviewer #1: (No Response)

Reviewer #2: -

Reviewer #3: The conclusions are discussed comprehensively in the context of the study findings. The limitations of the analysis are clearly described and addressed in sufficient detail and the authors adequately adress the public health relevance of the findings

**********

**Editorial and Data Presentation Modifications?**

Use this section for editorial suggestions as well as relatively minor modifications of existing data that would enhance clarity. If the only modifications needed are minor and/or editorial, you may wish to recommend “Minor Revision” or “Accept”.

Reviewer #1: (No Response)

Reviewer #2: -

Reviewer #3: There are no additional editorial or data presentation modifications required from my perspective. The figures and tables are clearly presented, well organized, and sufficiently detailed to support the manuscript in its current form.

**********

**Summary and General Comments**

Use this section to provide overall comments, discuss strengths/weaknesses of the study, novelty, significance, general execution and scholarship. You may also include additional comments for the author, including concerns about dual publication, research ethics, or publication ethics. If requesting major revision, please articulate the new experiments that are needed.

Reviewer #1: (No Response)

Reviewer #2: No new comments

Reviewer #3: The authors have addressed all questions and concerns raised in the review report in a detailed and comprehensive manner. The responses provided were clear, well explained, and adequately justified. The corresponding sections of the manuscript were appropriately revised and adapted accordingly and the revised version has improved in clarity and quality.

**********

PLOS authors have the option to publish the peer review history of their article (what does this mean?). If published, this will include your full peer review and any attached files.

If you choose “no”, your identity will remain anonymous but your review may still be made public.

**Do you want your identity to be public for this peer review?**  For information about this choice, including consent withdrawal, please see our Privacy Policy.

Reviewer #1: No

Reviewer #2: No

Reviewer #3: **Yes:**  Julia Meyer

# Editor report: Acceptance letter

**DOI:** 10.1371/journal.pntd.0014482.r004

**Authors:** Krystyna Cwiklinski, Marc Hübner, Krystyna Cwiklinski, Marc Hübner

Dear Ms. Dupper,

We are delighted to inform you that your manuscript, "Flubendazole 2.0: designing a large field trial in dogs for a challenging setting," has been formally accepted for publication in PLOS Neglected Tropical Diseases.

We have now passed your article onto the PLOS Production Department who will complete the rest of the publication process. All authors will receive a confirmation email upon publication.

The corresponding author will soon be receiving a typeset proof for review, to ensure errors have not been introduced during production. Please review the PDF proof of your manuscript carefully, as this is the last chance to correct any scientific or type-setting errors. Please note that major changes, or those which affect the scientific understanding of the work, will likely cause delays to the publication date of your manuscript. Note: Proofs for Front Matter articles (Editorial, Viewpoint, Symposium, Review, etc...) are generated on a different schedule and may not be made available as quickly.

Soon after your final files are uploaded, the early version of your manuscript will be published online unless you opted out of this process. The date of the early version will be your article's publication date. The final article will be published to the same URL, and all versions of the paper will be accessible to readers.

For Research Articles, you will receive an invoice from PLOS for your publication fee after your manuscript has reached the completed accept phase. If you receive an email requesting payment before acceptance or for any other service, this may be a phishing scheme. Learn how to identify phishing emails and protect your accounts at https://explore.plos.org/phishing.

Thank you again for supporting open-access publishing; we are looking forward to publishing your work in PLOS Neglected Tropical Diseases.

Best regards,

Shaden Kamhawi

co-Editor-in-Chief

PLOS Neglected Tropical Diseases

Paul Brindley

co-Editor-in-Chief

PLOS Neglected Tropical Diseases
